# SOX5 inhibition overcomes PARP inhibitor resistance in BRCA-mutated breast and ovarian cancer

**DOI:** 10.1038/s41419-025-07660-7

**Published:** 2025-04-24

**Authors:** Mithun Ghosh, Min Sil Kang, Nar Bahadur Katuwal, Sa Deok Hong, Seong Min Park, Seul-Gi Kim, Seung Ryeol Lee, Yong Wha Moon

**Affiliations:** 1 Department of Biomedical Science, The Graduate School, CHA University, Seongnam-si, 13488 Republic of Korea; 2 Department of Internal Medicine, Hematology and Oncology, CHA Bundang Medical Center, CHA University, Seongnam-si, 13496 Republic of Korea; 3 Department of Urology, CHA Bundang Medical Center, CHA University, Seongnam-si, 13496 Republic of Korea

**Keywords:** Breast cancer, Ovarian cancer

## Abstract

Poly (ADP-ribose) polymerase (PARP) inhibitors are effective in cells with homologous recombination (HR) deficiency, including BRCA1/2 mutation. However, PARP inhibitors remain a therapeutic challenge in breast and ovarian cancer due to inevitably acquired resistance in most cases. Therefore, strategies to overcome PARP inhibitor resistance are unmet clinical need. SRY-box transcription factor 5 (SOX5) plays a crucial role in development of various cancers but the role of SOX5 in PARP inhibitor resistance is poorly understood. This study identified SOX5 as a potential biomarker associated with PARP inhibitor resistance and addressed potential treatment strategies to overcome PARP inhibitor resistance using the olaparib-resistant preclinical model. We observed that SOX5 was significantly upregulated in olaparib-resistant cells and contributed to PARP inhibitor resistance by upregulating DNA repair pathway genes. Ectopic SOX5 overexpression contributed to PARP inhibitor resistance by suppressing DNA double-strand breaks (DSBs) in BRCA-mutated breast and ovarian cancer. SOX5 small interfering RNA combined with olaparib sensitized olaparib-resistant cells and suppressed the growth of olaparib-resistant xenografts in mice via increased DSBs represented by ɣH2AX formation. Mechanistically, SOX5 directly interacted with yes-associated protein 1 (YAP1) and promoted its nuclear translocation by suppressing the Hippo pathway. YAP1, in association with TEA domain family members (TEAD), upregulated HR-related gene expression and conferred PARP inhibitor resistance. Furthermore, the clinical relevance of SOX5 as a therapeutic target was supported by a significant association between SOX5 overexpression and poor prognosis in ovarian cancer on public mRNA microarray data sets. Therefore, we propose SOX5 as a promising therapeutic target for overcoming PARP inhibitor resistance in BRCA1/2-mutated breast and ovarian cancer.

## Introduction

BRCA1 and BRCA2 (BRCA1/2) are crucial genes of the DNA damage response (DDR) pathway involved in homologous recombination (HR) repair (HRR) to resolve DNA double-strand breaks (DSBs) [1]. Hence, BRCA1/2-mutated cells are synthetically lethal to poly (ADP-ribose) polymerase (PARP) inhibitors due to DNA DSB accumulation resulting from irreparable single-strand breaks [2]. Ovarian cancers show the highest prevalence of BRCA1/2 mutations at 21% [3] but ~5% in several other cancers, such as breast [4], prostate [5], non-small cell lung [6], pancreatic [7], bile duct [8], and bladder [9]. Considerate portions of patients with BRCA1/2 mutations across various cancers can be potential candidates for PARP inhibitor therapy.

In BRCA1/2-mutated or HR-deficient (HRD) ovarian cancer, olaparib or niraparib maintenance therapy after first-line paclitaxel/carboplatin has demonstrated a significant increase in progression-free survival (PFS) in SOLO1 [10] and PRIMA [11] trials, respectively. In breast cancer with BRCA1/2 mutation, olaparib or talazoparib has significantly prolonged PFS in OLYMPIAD [12] and EMBRACA [13] trials of metastatic setting, respectively, and olaparib has significantly improved invasive disease-free survival and overall survival (OS) in OLYMPIA trial of adjuvant setting [14, 15]. These PARP inhibitors have been approved for the aforementioned indications by the United States Food and Drug Administration. Apart from ovarian and breast cancers, other BRCA1/2-mutated cancers, including prostate [16], pancreatic [7], and small cell lung [6], have demonstrated clinical efficacy with PARP inhibitor therapy.

Despite the initial durable response of PARP inhibitors in BRCA1/2-mutated cancers, most patients develop PARP inhibitor resistance [17]. Based on preclinical studies, acquired PARP inhibitor resistance develops by several mechanisms: (i) restoration of the HRR capacity directly through reversion mutation of HRR genes [e.g., BRCA1/2 and the DNA repair protein RAD51 homolog 1 (RAD51)] or indirectly through upregulation of oncogenic signaling that activates HRR gene expression, (ii) replication fork stabilization, (iii) upregulation of drug efflux pumps, (iv) mutations in catalytic or drug-binding domain of PARP, and (v) increased PARylation [18]. However, no strategies to overcome PARP inhibitor resistance have been clinically established. Therefore, studies should endeavor to reveal novel targets to overcome PARP inhibitor resistance.

An RNA sequencing (RNA-seq) analysis comparing parental BRCA2-mutated cancer cells and olaparib-resistant cells identified SRY-box transcription factor 5 (SOX5) as one of the top candidate genes upregulated in olaparib-resistant cells compared to the corresponding parental cells. Therefore, it was speculated that SOX5 may promote PARP inhibitor resistance. SOX5 is a member of the SOX family of transcription, which plays crucial roles in embryonic development cell fate determination and maintenance of normal physiological conditions [19, 20]. However, aberrant SOX5 expression is associated with the progression of various cancers [20–24]. Furthermore, SOX5-induced epithelial-to-mesenchymal transition has been demonstrated in several studies [22, 25]. However, to the best of our knowledge, no study has reported that SOX5 is associated with PARP inhibitor resistance. Therefore, these motivated us to investigate whether SOX5 is involved in PARP inhibitor resistance.

Based on the inevitable occurrence of PARP inhibitor resistance and a lack of established therapies to overcome resistance, a preclinical model with olaparib-resistant breast and ovarian cancer was developed to study the mechanisms of PARP inhibitor resistance. This screening assay discovered SOX5 as a top candidate gene, demonstrated how SOX5 is involved in PARP inhibitor resistance, and provided a scientific rationale for SOX5 as a promising therapeutic target for overcoming PARP inhibitor resistance.

## Materials and methods

### Drugs

Olaparib (cat# CS-0075) and talazoparib (cat# CS-0937) were purchased from ChemScene (Monmouth, NJ, USA) and dissolved in dimethyl sulfoxide (DMSO; Sigma-Aldrich, St. Louis, MO, USA).

### Cell culture

SNU-251, BRCA1-mutated, an ovarian cancer cell line (endometrioid carcinoma), was purchased from the Korean Cell Line Bank (Seoul, South Korea). BT-474, BRCA2-mutated, an estrogen receptor-positive/human epidermal growth factor receptor 2-positive breast cancer cell line (invasive ductal carcinoma), was purchased from the American Type Culture Collection (Manassas, VA, USA). The characteristics of BT-474 and SNU-251 cell lines are summarized in Table S1. All cell lines were cultured in RPMI 1640 medium (Welgene, Inc., Daegu, South Korea) containing 10% heat-inactivated fetal bovine serum (FBS; Welgene) and 1% penicillin/streptomycin solution (Welgene). All cells were maintained in a humidified atmosphere containing 5% CO_2_ at 37 °C.

### Olaparib-resistant cell line establishment

Olaparib-resistant cell lines were established by gradually increasing the olaparib concentration, starting from 1/15 of half-maximal inhibitory concentration (IC_50_), which were 4.8 and 3.7 µM for BT-474 and SNU-251 cell lines, respectively. Fresh medium and medium containing drugs were replenished every 3–4 days. Once cells could proliferate freely in the drug-containing medium, the drug concentration was increased. After 8–9 months of this process, olaparib-resistant cells were established and exhibited a 4.5–5-fold increase in the IC_50_ concentration. Those cells were termed BT-474-OR and SNU-251-OR in this study, indicating olaparib-resistant BT-474 and SNU-251 cells, respectively. Olaparib-resistant cells were maintained in 1/10 of IC_50_ of olaparib. Before performing any experiment, the drug was washed out before 48 h and maintained in a fresh medium.

### Cell viability assay

Cell viability was measured using the thiazoyl blue tetrazolium bromide (MTT; Sigma, St. Louis, MO, USA) colorimetric assay. In detail, 1500–1800 cells were seeded into a 96-well plate and incubated overnight. Cells were treated with different concentrations of olaparib and talazoparib and a fixed concentration of SOX5 small interfering RNA [(siRNA) cat# 4392420, Ambion; Thermo Fisher Scientific, Waltham, MA, USA] for 72 h. The MTT solution (10%) was added to each well and incubated for 4 h at 37 °C. After 4 h incubation, the medium was discarded, and DMSO was added and incubated at room temperature (RT) for 30 min to dissolve the formazan. A microplate reader (Multiskan FC; Thermo Fisher Scientific) was used to detect the optical density at 540 nm. CompuSyn software (ComboSyn, Inc., Paramus, NJ, USA) was used to evaluate the IC_50_ and combination index (CI) values, where CI < 1, CI > 1, and CI = 1 were considered synergism, antagonism, and additive effects, respectively.

### Western blot analysis

Harvested cells were washed with phosphate-buffered saline (PBS) and lysed in radioimmunoprecipitation assay (cat# 89901, Thermo Fisher Scientific) buffer which containing a protease inhibitor cocktail (cat# 11873580001, Roche, Basel, Switzerland) and phosphatase inhibitor (cat# 1862495, Thermo Fisher Scientific) for 1 h at 4 °C. Whole protein lysates were separated using sodium dodecyl sulfate-polyacrylamide gel electrophoresis and transferred onto polyvinylidene difluoride membranes (cat# IPVH00010, Millipore, Burlington, MA, USA). The membranes were blocked for 1 h at RT with 5% skim milk in Tris-buffered saline/0.1% Tween 20. Blots were incubated with primary antibody overnight at 4 °C and horseradish peroxidase-conjugated secondary antibody for 1 h. The bands were detected using a Detection Reagent 1 peroxide solution (cat# 1859700, Thermo Fisher Scientific) and a Detection Reagent 2 luminol enhancer Solution (cat# 1859697, Thermo Fisher Scientific). The images were taken using GeneSys software.

To conduct the coimmunoprecipitation assay, cells were lysed in NETN lysis buffer [250 mM NaCl (pH 8.0), 5 mM Tris-HCl, 5 mM EDTA, and 0.5% NP-40 (Igepal CA-630; Sigma-Aldrich)] containing protease and phosphatase inhibitor cocktail. Total protein lysates (1000 μg) were immunoprecipitated with anti-YAP or anti-SOX5 (1:50) antibody overnight with 30 µL Protein A/G beads (cat# 20421, Thermo Fisher Scientific). Normal rabbit IgG isotypes (cat# 3900, Cell Signaling Technology, MA, USA) were used as a negative control. The bands were detected as mentioned above. A list of primary and secondary antibodies is provided in Table S2.

### Nuclear and cytoplasmic protein extraction

Nuclear and cytoplasmic protein separation was performed using the Subcellular Protein Fractionation Kit for cultured cell reagents (cat# 78840, Thermo Fisher Scientific) according to the manufacturer’s instructions. Ice-cold cytoplasmic extraction buffer containing protease inhibitors was added to the cell pellet, incubated at 4 °C for 10 min, and centrifuged at 500 × *g* for 5 min, and the supernatant was immediately collected (cytoplasmic extract). An ice-cold membrane extraction buffer containing protease inhibitors was added to the pellet, incubated at 4 °C for 10 min, and centrifuged at 3 000 × *g* for 5 min. The supernatant was collected. An ice-cold nuclear extraction buffer containing protease inhibitors was added to the pellet and vortexed on the highest setting for 15 s. Cells were incubated at 4 °C for 30 min and centrifuged at 5000 × *g* for 5 min, and the supernatant (soluble nuclear extract) was collected. The samples were subjected to Western blot analysis, as mentioned above.

### Quantitative real-time polymerase chain reaction (qRT-PCR)

Cells were washed with PBS, and total mRNA was extracted from cells using TRIzol reagent (cat# 15596026, Ambion, Life Technologies, Carlsbad, CA, USA). cDNA was synthesized from total mRNA using the Takara Prime Script First-Strand cDNA Synthesis kit (cat# 6110 A, Takara Bio, Inc., Shiga, Japan). qRT-PCR was conducted using a Power-up SYBR Green Master Mix (cat# A25741, Thermo Fisher scientific) and an ABI StepOne Real-time PCR System (Applied Biosystems, Warrington, UK). Glyceraldehyde 3-phosphate dehydrogenase was used as endogenous control. The 2^−ΔΔct^ method was used to calculate gene expression. A list of primers is included in Table S3.

### Apoptosis assay

An apoptosis assay was carried out using flow cytometry. After detaching via trypsinization, cells were washed twice with PBS and centrifuged at 500 RCF. Cells (1 × 10^6^/mL) were resuspended in Annexin V binding buffer (cat# 422201, BioLegend, San Diego, CA, USA). A 100 µL cell suspension was transferred into a microcentrifuge tube and stained with 5 µL APC Annexin V (cat# 640920, BioLegend) and 10 µL propidium iodide (Invitrogen, MA, USA). The samples were incubated for 15 min at RT, and 400 µL Annexin V binding buffer was added to each tube. Apoptotic cells were analyzed via flow cytometry (CytoFLEX, Beckman Coulter, Brea, CA, USA).

### Immunocytochemistry (ICC)

Cells were washed with PBS and fixed with 4% paraformaldehyde for 15 min on ice. Cells were washed thrice with PBS and incubated for 10 min at RT in the presence of permeabilization buffer (containing 0.2% Triton-X in PBS). After washing with PBS, cells were blocked using a blocking buffer containing 5% bovine serum albumin and 0.3% Triton-X in PBS for 20 min at RT. Cells were incubated for 2 h at RT with the primary antibody and washed thrice with PBS. Fluorescently labeled secondary antibody (Alexa Fluor 488 conjugated) was added and incubated for 30 min at RT in the dark. The Vectashield mounting medium containing 4′,6-diamidino-2-phenylindole (DAPI, Vector Laboratories, Inc., Burlingame, CA, USA) was used to mount the cells. Images were captured using a Zeiss 510 fluorescence microscope (Zeiss, Oberkochen, Germany).

### Small interfering RNA (siRNA) transfection

RNAiMAX reagent (cat# 13778-030, Invitrogen, MA, USA) was used to conduct the siRNA transfection according to the manufacturer’s protocol. Cells were seeded into a 60 mm culture dish at ~60% to 80% confluency during transfection and incubated overnight. Lipofectamine RNAiMAX reagent and SOX5 siRNA (cat# 4392420, Ambion, Thermo Fisher Scientific) were diluted separately in Opti-MEM® Reduced-Serum Medium (cat# 31985070, Thermo Fisher Scientific). Diluted siRNA and the diluted Lipofectamine RNAiMAX reagent were mixed and incubated for 10–15 min at RT. Cells were treated with the siRNA-lipid complex and incubated for 48–72 h at 37 °C in a humidified CO_2_ incubator. After incubation, cells were harvested, and Western blot analysis was performed to evaluate the transfection efficiency of siRNA.

### Plasmids and ectopic overexpression studies

The SOX5 overexpression vector, (cat# RC224228) and the respective empty vector (cat# PS100001) were purchased from Origene (Rockville, MD, USA). The pEGFP-C3-hYAP1 (Addgene plasmid #17843) overexpression vector was purchased from Addgene (Watertown, MA, USA). Transfection was performed through CalFectin™ Mammalian DNA transfection reagent (cat# SL100478, SignaGen Laboratories, Frederick, MD, USA) according to the manufacturer’s protocol with little modification. Cells (5 × 10^5^) were seeded in a 60 mm cell culture disk and incubated overnight. The complete culture medium with serum and antibiotics was freshly added to the plates before transfection. A 1 µg plasmid DNA was diluted gently into 250 µL FBS and antibiotic-free DMEM and vortex. A 5 µL CalFectin™ reagent was added immediately directly into the 250 µL diluted DNA solution and mixed gently by pipetting up and down three to four times. The mixture was incubated for 10–15 min at RT to allow the CalFectin™/DNA complex to form. The CalFectin™/DNA complex was added dropwise to the medium, and the mixture was homogenized by gently swirling the plate. After 48 h incubation, cells were collected, and overexpression was confirmed by Western blot analysis.

### Fluorescence imaging

Transfected cells were fixed with 4% paraformaldehyde for 15 min on ice, followed by three washes with PBS. Cells were then permeabilized by incubation for 10 min at RT in permeabilization buffer. Subsequently, cells were mounted using Vectashield mounting medium containing 4′,6-diamidino-2-phenylindole (DAPI, Vector Laboratories, Inc., Burlingame, CA, USA). Fluorescence images were acquired using a Zeiss 510 fluorescence microscope (Zeiss, Oberkochen, Germany).

### Sanger sequencing

Sanger sequencing for BRCA1 in SNU-251 and SNU-251-OR cells were conducted by Macrogen Inc. (Seoul, South Korea). Briefly, Genomic DNA was extracted using InstaGene Matrix (cat#732-6030, BIO-RAD, Hercules, CA, USA) with a DNA concentration of 20–100 ng per reaction. PCR amplification was performed using MBAE-110 Axen™ H Taq PCR Master Mix (Roche, Basel, Switzerland) on the DNA Engine Tetrad 2 Peltier Thermal Cycler (BIO-RAD, Hercules, CA, USA). PCR products were purified with ExoSAP-IT™ Express PCR Product Cleanup (Thermo Fisher scientific). Sequencing reactions were prepared using BigDye® Terminator v3.1 Cycle Sequencing Kit (Applied Biosystems, Waltham, MA, USA) and analyzed on the ABI PRISM 3730XL Analyzer (96-capillary type). Sequence data were processed using Variant Reporter Software version 2.1 (Applied Biosystems, Waltham, MA, USA).

### RNA-seq

To analyze differentially expressed genes (DEGs) between BRCA-mutated parental cells (BT-474) and olaparib-resistant cells (BT-474-OR), RNA-seq was conducted. The total RNA concentration was measured by Quant-IT RiboGreen (cat# R11490, Invitrogen, MA, USA). To analyze the total RNA integrity, the samples were run on the TapeStation RNA ScreenTape (cat# 5067-5576, Agilent Technologies, Santa Clara, CA, USA). Only high-quality RNA preparations, with RIN > 7.0, were used for RNA library construction. An independent library was prepared with 1 μg total RNA for each sample by Illumina TruSeq Stranded mRNA Sample Prep Kit (Illumina, Inc., San Diego, CA, USA). The poly(A)-containing mRNA molecules were purified using poly(T)-attached magnetic beads. After purification, mRNA was fragmented into small pieces using divalent cations under elevated temperature. The cleaved RNA fragments were copied into first-strand cDNA using SuperScript II reverse transcriptase (cat# 18064014, Invitrogen, MA, USA) and random primers followed by second-strand cDNA synthesis using DNA polymerase I, RNase H, and dUTP. The products were purified and enriched with PCR to create the final cDNA library. The libraries were quantified using KAPA Library Quantification kits for Illumina Sequencing platforms according to the qPCR Quantification Protocol Guide (cat# KK4854, Kapa Biosystems, Wilmington, MA, USA) and qualified using the TapeStation D1000 ScreenTape (cat# 5067-5582, Agilent Technologies, Santa Clara, CA USA). Indexed libraries were submitted to an Illumina NovaSeq (Illumina, Inc., CA, USAS), and the paired-end (2 × 100 bp) sequencing was performed by Macrogen, Inc. (Seoul, South Korea).

### Animal studies

The animal experimental procedure was performed as described earlier [26]. BALB/c nude mice were purchased from JA Bio, Inc. (Suwon-si, Gyeonggi-do, Republic of Korea) and kept in a specific pathogen-free environment for 1 week to acclimatize at CHA University (Seongnam-si, Republic of Korea). To induce a tumor, BT-474-OR cells (1 × 10^7^ per mouse in 0.05 mL PBS) were mixed with 0.05 mL Matrigel (cat# 354248, Corning, NY, USA) and injected subcutaneously into the mammary fat pad. For estrogen supplement, estrogen valerate (3 µg/mouse) was subcutaneously injected weekly. When tumor volume reached 70–80 mm^3^, mice were randomized into four groups containing five per group. Mice were treated with control siRNA 5 µg/mouse (group 1), olaparib 50 mg/kg twice daily (group 2), SOX5 siRNA 5 µg/mouse (group 3, Ambion® In Vivo siRNA, Thermo Fisher Scientific), and SOX5 siRNA with olaparib (group 4). siRNAs were prepared using in vivo-jetPEI (cat# 201-10 G, Polyplus) transfection reagent according to the manufacturer’s protocol (N/P ration 6). siRNA was injected intratumorally every 3 days seven times, and olaparib was administered orally. Tumor sizes were measured thrice a week using a Vernier caliper. Tumor volume was measured using the formula: tumor length × tumor width × 0.5. All mice were sacrificed, and tumors were preserved for further analysis.

### Terminal deoxynucleotidyl transferase dUTP nick end labeling (TUNEL) assay

TUNEL assay was performed to identify apoptotic cells in xenograft tissues according to the manufacturer’s (cat# S7101, Merck, MA, USA) protocol as mentioned previously [27]. Tissue sections were deparaffinized and hydrated. The sections were treated with TUNEL and hematoxylin for the indicated times. To quantify TUNEL assay results, the number of positively stained cells was counted under a light microscope in five random nonoverlapping 400× microscopic fields for each tissue section.

### Public gene expression profiling data sets in patients with ovarian cancer

To evaluate whether SOX5 mRNA expression levels were associated with prognosis in patients with ovarian cancer, all available (seven data sets were available for Kaplan–Meier plot analysis under the Affymetrix ID 1569638_at) independent public mRNA expression data sets of primary debulked ovarian cancer (GSE26193, GSE27651, GSE30161, GSE19829, GSE18520, GSE63885, and GSE9891) were analyzed using the online platform Kaplan–Meier plotter [28] (https://kmplot.com/analysis/) without downloading processed raw data. The U133 GeneChip™ Human Genome U133 Plus 2.0 Array was used for all GSE data sets. Affymetrix ID 1569638_at was selected for SOX5. SOX5 mRNA expression levels were divided into two groups, “SOX5-High” and “SOX5-Low,” with the auto-select best cutoff. An optimal cutoff point for the normalized intensity of SOX5 mRNA was determined using a minimum *p* value approach in predicting OS [29].

### Statistical analysis

All statistical analyses were performed using SPSS version 20 (IBM SPSS Statistics 20; Armonk, NY, USA). Student’s *t*-test was conducted to compare the two groups in the MTT assay, Western blot analysis, qRT-PCR, apoptosis assay, ICC, tumor growth inhibition and TUNEL assay. OS was defined as the time from diagnosis of ovarian cancer to death from any cause or the last date at which the patient was known to be alive (censoring time). OS was calculated using the Kaplan–Meier method. A log-rank test was used to compare OS between groups. All *p* values were two-sided, and *p* < 0.05 was considered statistically significant.

## Results

### Generation and confirmation of olaparib-resistant cell lines

**Fig. 1:**
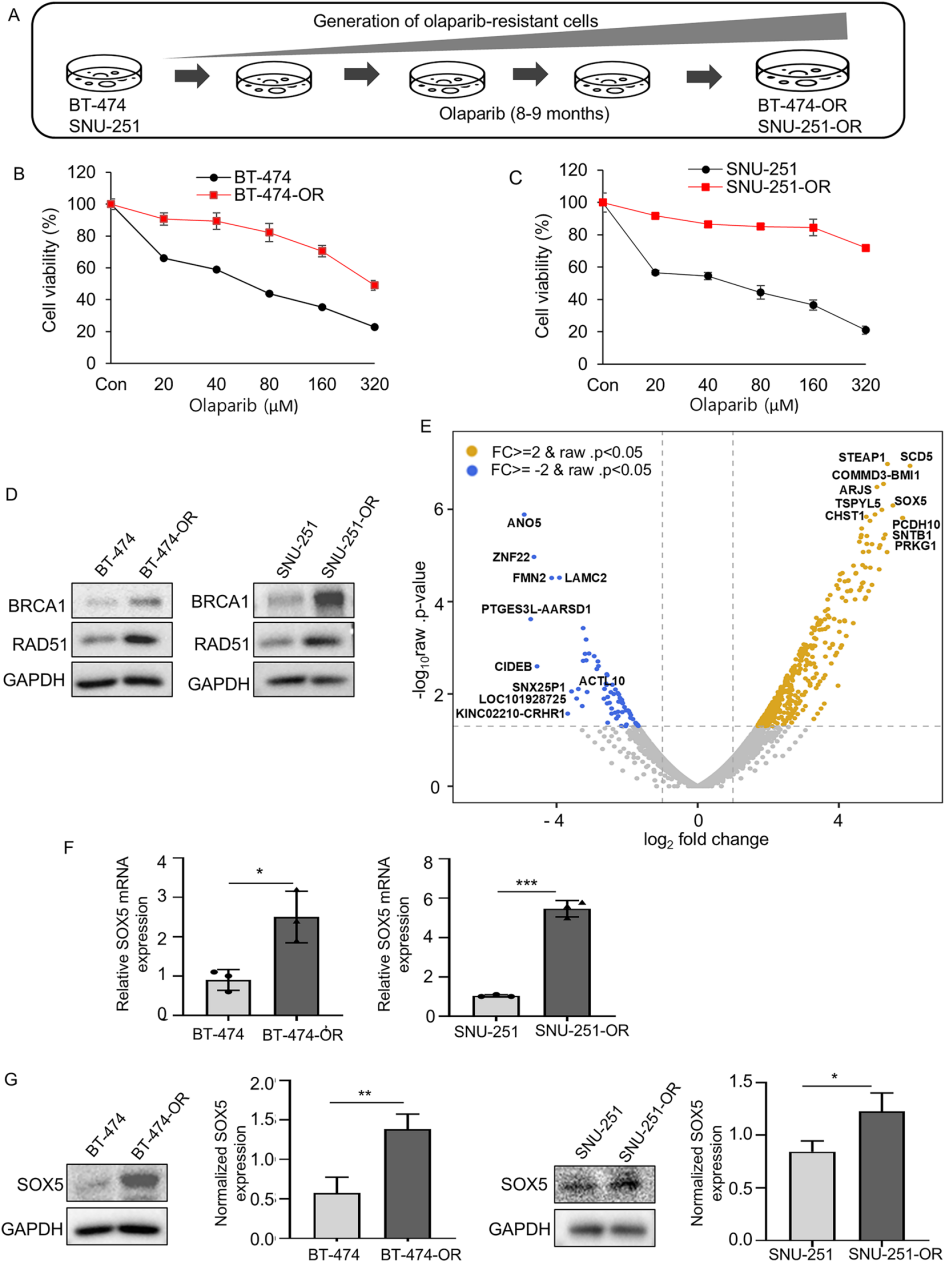
Generation and confirmation of olaparib-resistant cell lines and SOX5 is upregulated in olaparib-resistant cell. **A** Schematic diagram of olaparib-resistant cell generation. BT-474 and SNU-251 cells were continuously treated with a gradually increasing concentration of olaparib for 8–9 months. The resistant cells were named BT-474-OR and SNU-251-OR. **B**, **C** Cell viability was measured by MTT assay in parental cells (BT-474, SNU-251) and olaparib-resistant cells (BT-474-OR, SNU-251-OR). Cells were treated with various concentrations of olaparib for 72 h. **D** Western blot analysis showed that expression of BRCA1 and RAD51 was upregulated in olaparib-resistant cells (BT-474-OR, SNU-251-OR) as compared to their respective parental cells (BT-474, SNU-251). **E** Volcano plot showed DEGs in BT-474-OR compared with BT-474 from RNA sequencing analysis. Blue indicated downregulated genes and light yellow indicated upregulated genes. FC, fold change. SOX5 was highly upregulated in BT-474-OR cells. **F** The relative mRNA expression was determined by qRT-PCR. SOX5 mRNA expression was upregulated in the olaparib-resistant cells (BT-474-OR, SNU-251-OR) as compared to parental cells (BT-474, SNU-251). Data were presented as mean ± SD of triplicate experiments. *P* values were calculated by Student’s *t*-test, indicating *p < 0.05, ***p < 0.001. **G** Western blot analysis exhibited the expression level of SOX5 in olaparib-resistant cells (BT-474-OR, SNU-251-OR) cells versus parental cells (BT-474, SNU-251). SOX5 was significantly upregulated in olaparib-resistant cells as compared to parental cells. Bar graphs exhibited normalized SOX5 expressions from 3 independent experiments. SOX5 expression was normalized with GAPDH. Data were presented as mean ± SD from 3 independent experiments. *P* values were calculated by Student’s *t*-test, indicating *p < 0.05, **p < 0.01. GAPDH was used as loading control.

Olaparib-resistant BRCA-mutated breast and ovarian cancer cell lines were generated as described in the Materials and Methods (Fig. 1A). Cell proliferation assay showed a significant increase in the IC_50_ of olaparib-resistant cells (320 µM in BT-474-OR and 350 µM in SNU-251-OR) compared to their corresponding parental cells (72 µM in BT-474 and 56 µM in SNU-251; Fig. 1B, C). Olaparib-resistant cells also showed cross-resistance with another PARP inhibitor, talazoparib (Supplementary Fig. S1A, B). Based on various reports, restoring HR activity is one of the PARP inhibitor resistance mechanisms [18]. Therefore, expression of HR-related genes, such as BRCA1 and RAD51, was analyzed in olaparib-resistant cells and their corresponding parental cells. Intriguingly, BRCA1 and RAD51 expression was upregulated in both resistant cells (Fig. 1D). SNU-251 exhibits a nonsense mutation in exon 23 of BRCA1 gene, leading to the substitution of tryptophan (TGG) at codon 1815 with a stop codon (TGA). This mutation results in a truncated BRCA1 protein that lacks the final 44 amino acids at the C-terminus [30]. As SNU-251-OR cells exhibited BRCA1 protein overexpression, we aimed to determine whether they had acquired a BRCA1 reversion mutation. Sequencing analysis confirmed that SNU-251-OR cells retained the same mutation as the SNU-251 parental cells (Supplementary Fig. S1C).

### SOX5 is upregulated in olaparib-resistant cells

To analyze DEGs in olaparib-resistant cells, RNA-seq analysis was performed in parental (BT-474) and resistant (BT-474-OR) cells. The volcano plot (Fig. 1E) represented DEGs by BT-474-OR compared to BT-474 cells (BT-474-OR/BT-474), and the top 10 upregulated and top 10 downregulated DEGs were indicated. Additionally, a list of 20 upregulated and downregulated genes is provided in Table S4. After a literature review, aberrant SOX5 expression was reported to play a critical role in the progression of various cancers, such as breast, lung, colorectal, melanoma, lymphoma, and hepatocellular carcinoma [20–23]. However, based on our knowledge, whether SOX5 plays any role in PARP inhibitor resistance development has not been reported. As RNA-seq results showed that SOX5 is one of the top upregulated genes in olaparib-resistant cells, SOX5 was chosen as a potential candidate gene to determine whether it plays any specific role in PARP inhibitor resistance development. SOX5 mRNA and protein expression was significantly upregulated in olaparib-resistant cells (BT-474-OR and SNU-251-OR) by qRT-PCR and Western blot analysis, respectively, compared to their respective parental cells (BT-474 and SNU-251; Fig. 1F, G).

### SOX5 inhibition synergistically suppresses olaparib-resistant cells in combination with olaparib (or talazoparib) through DNA DSB

**Fig. 2:**
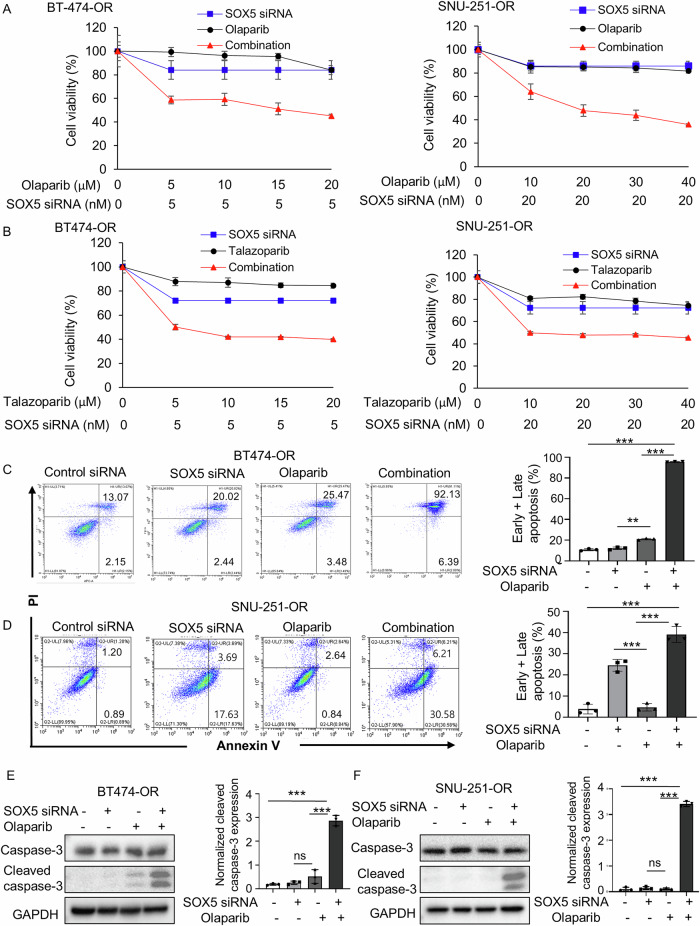
SOX5 inhibition synergizes with olaparib (or talazoparib) in olaparib-resistant cell. **A**, **B** Cell viability (MTT) assay of BT-474-OR and SNU-251-OR cells. Cells were treated with SOX5 siRNA, various concentrations of olaparib (or talazoparib) and their combination and incubated for 72 h. **C**, **D** Apoptosis assay conducted by flow cytometry. Representative scatter plots of PI (y-axis) and annexin V (x-axis) are shown. Cells were treated with SOX5 siRNA (5 nmol in BT474-OR and 20 nmol in SNU-251-OR), olaparib (10 µM) and their combination for 72 h. The bar graphs depicted the average of total apoptotic cells of 3 independent experiments. *P* values were calculated by Student’s *t*-test, indicating **p < 0.01, ***p < 0.001. Data are presented as mean ± SD from 3 independent experiments. **E**, **F** Western blot analysis showed the expressions of apoptosis marker, caspase-3, and cleaved caspase-3. Cells were treated with SOX5 siRNA (5 nmol in BT474-OR and 20 nmol in SNU-251-OR), olaparib (5 µM) and their combination for 72 h. The bar graphs showed the normalized expression of cleaved caspase-3 based on densitometric analysis from 3 independent experiments. *P* values were calculated by Student’s *t*-test, indicating ***p < 0.001. ns, not significant. Data are presented as mean ± SD from 3 independent experiments. GAPDH was used as a loading control.

The combination of olaparib (or talazoparib) and SOX5 siRNA synergistically (CI < 1; Supplementary Fig. S2A–D) suppressed cell proliferation compared to the monotherapy (Fig. 2A, B) in both cell lines. In addition, the combination treatment of olaparib (or talazoparib) and SOX5 siRNA significantly increased the total apoptotic cells compared to olaparib or SOX5 siRNA alone (Fig. 2C, D, Supplementary Fig. S2E, F). Cleaved caspase-3 expression was increased after the combination treatment of olaparib (or talazoparib) and SOX5 siRNA (Fig. 2E, F, Supplementary Fig. S2G, H). Based on these results, it was hypothesized that the observed synergistic effects may be due to enhanced apoptosis by combination treatment.

**Fig. 3:**
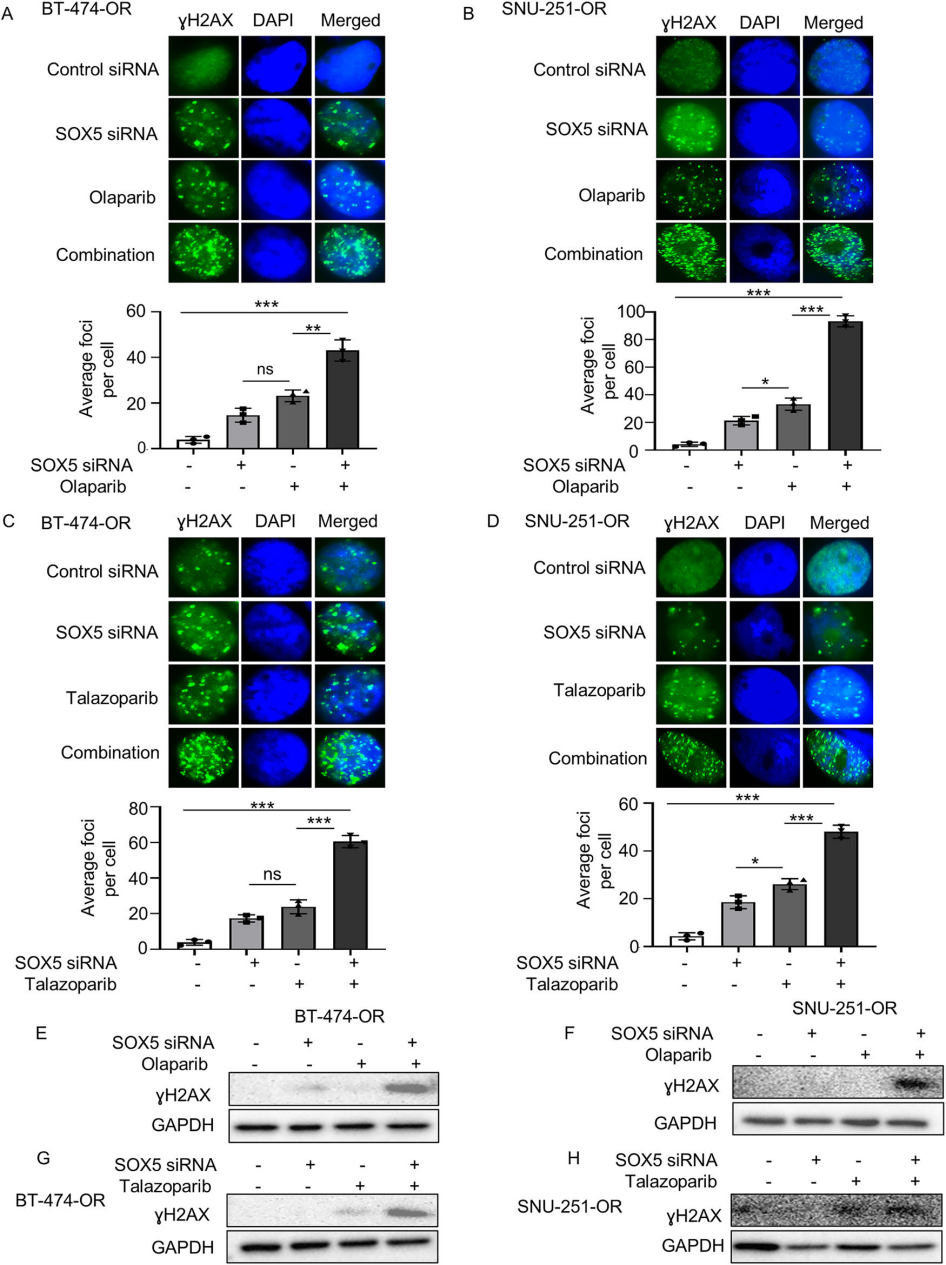
Increased DNA double strand-break could be the reason for synergistic effects between SOX5 inhibition and olaparib (or talazoparib). **A**–**D** Immunofluorescence images showed the ɣH2AX foci formation in BT-474-OR and SNU-251-OR cells. Cells were treated with SOX5 siRNA (5 nmol in BT474-OR and 20 nmol in SNU-251-OR) and 5 µM of olaparib (or talazoparib, 5 µM) and their combination for 72 h. The images were taken at 100x magnification. The bar graphs depicted the average foci number per cell. Data were averaged from three independent experiments, with 100 cells analyzed per condition in each experiment. Data are presented as mean ± SD. *P* values were calculated by Student’s *t*-test, indicating *p < 0.05, **p < 0.01, ***p < 0.001. ns, not significant. **E**–**H** The expression of ɣH2AX was evaluated by western blot analysis in BT-474-OR and SNU-251-OR cells. Cells were treated with SOX5 siRNA (5 nmol in BT474-OR and 20 nmol in SNU-251-OR), 5 µM of olaparib (or talazoparib, 5 µM) and their combination for 72 h. GAPDH was used as loading control.

To determine the reason behind the abovementioned apoptosis, an ICC assay was performed to analyze the ɣH2AX foci formation because olaparib (or talazoparib) is known to induce DNA DSBs. The combination of olaparib (or talazoparib) and SOX5 siRNA significantly exhibited profound DSBs than did SOX5 siRNA or olaparib (or talazoparib) alone (Fig. 3A–D). Furthermore, the γH2AX expression was remarkably increased in combination with olaparib (or talazoparib) and SOX5 siRNA in both olaparib-resistant cells (BT-474-OR and SNU-251-OR, Fig. 3E–H). Therefore, SOX5 inhibition combined with olaparib (or talazoparib) led to increased DSBs, which could be the reason for enhanced apoptosis and synergistic anticancer effects.

To further investigate BRCA1 activity in double-strand break repair through foci assembly, we conducted an ICC experiment. Our results showed that treatment with olaparib or talazoparib increased BRCA1 foci formation, suggesting its involvement in double-strand break repair and a potential mechanism of resistance. In contrast, combination treatment significantly reduced BRCA1 foci formation, indicating impaired DNA double-strand break repair and enhanced drug sensitivity (Supplementary Fig. S3A–D).

### Ectopic SOX5 overexpression leads to PARP inhibitor resistance

**Fig. 4:**
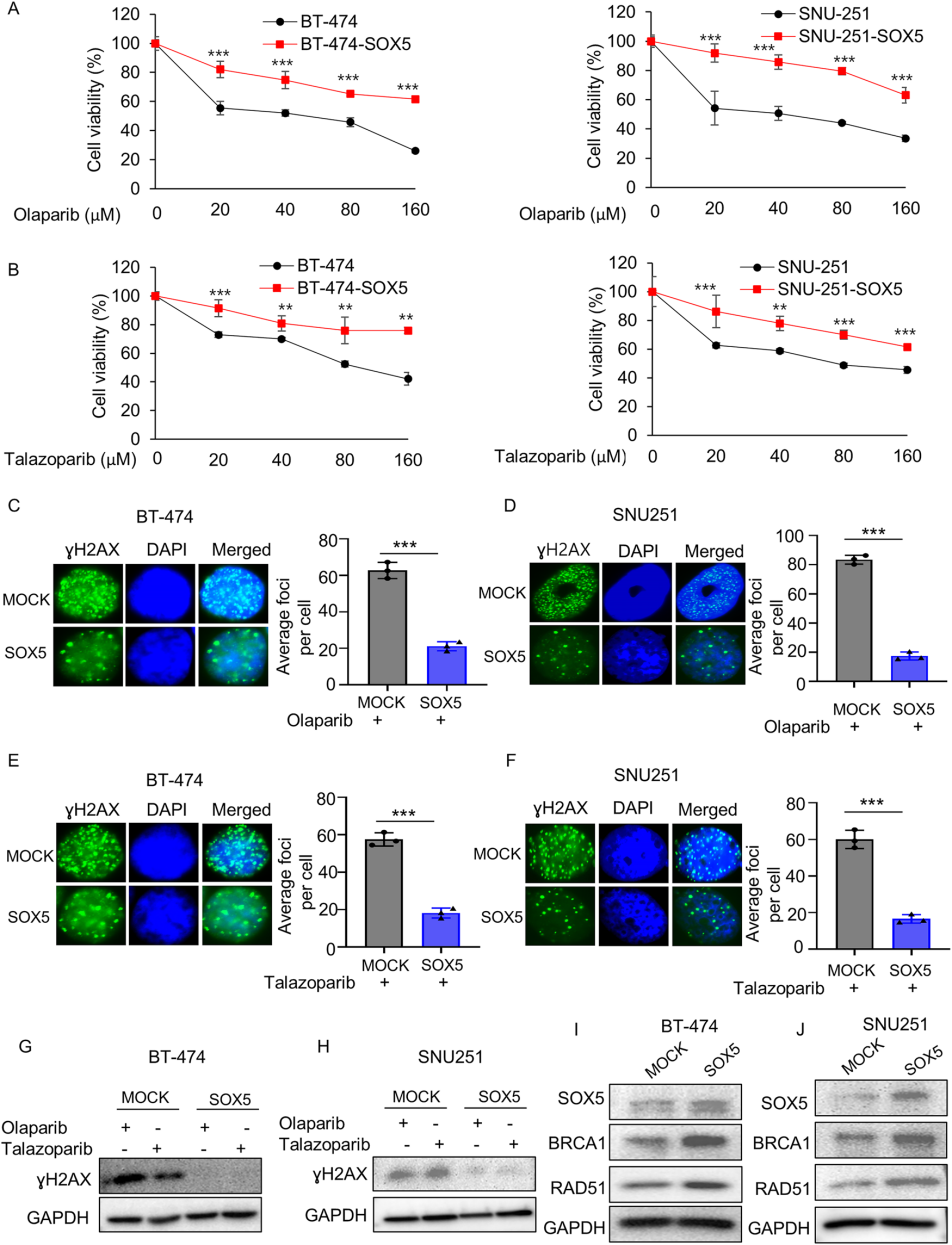
Ectopic overexpression of SOX5 leads to PARP inhibitor resistance. **A**, **B** MTT assay was used to measure the cell viability of BT-474 and SNU-251 cells before and after the ectopic overexpression of SOX5. Cells were treated with the indicated concentrations of olaparib or talazoparib for 72 h. Data shown here were the representative of 3 independent experiments. *P* values were calculated by Student’s *t*-test, indicating **p < 0.01, ***p < 0.001. **C**–**F** ɣH2AX foci were determined by Immunofluorescence assay in BT-474, SNU-251 parental cells and ectopically SOX5-overexpressed cells. Cells were treated with 5 µM of olaparib (or talazoparib, 5 µM) and incubated for 72 h. The images were taken at 100x magnification. The bar graphs depicted the average foci number per cell. Data were averaged from three independent experiments, with 100 cells analyzed per condition in each experiment. Data are presented as mean ± SD. *P* values were calculated by Student’s *t*-test, indicating ***p < 0.001. **G**, **H** Western blot analysis was carried out to determine the expression of ɣH2AX in BT-474, SNU-251 parental cells and ectopically SOX5-overexpressed cells. Cells were treated with olaparib (5 µM) and talazoparib (5 µM) for 72 h. GAPDH was used as loading control. **I**, **J** Western blot analysis showed that expression of BRCA1 and RAD51 was increased in SOX5-overexpressed cells. GAPDH was used as loading control.

Because SOX5 was upregulated in olaparib-resistant cells and its inhibition combined with PARP inhibitors showed synergistic anticancer effects, this study sought to determine whether SOX5 overexpression plays any role in PARP inhibitor resistance development and DSB repair. To do so, SOX5 was ectopically overexpressed in BT-474 and SNU-251 parental cells. The antiproliferative efficacy of olaparib and talazoparib decreased in both ectopically SOX5-overexpressed cells (Fig. 4A, B), suggesting that SOX5 is associated with PARP inhibitor resistance in those cells.

It was hypothesized that SOX5 overexpression may suppress DSBs, as opposed to SOX5 inhibition enhancing DSBs, as shown above. ICC results demonstrated that SOX5 overexpression significantly inhibited γH2AX foci formation in the presence of olaparib (or talazoparib; Fig. 4C–F), indicating DSB suppression. Western blot analysis results confirmed again that the induction of ɣH2AX protein by olaparib (or talazoparib) remarkably decreased in SOX5-overexpressed cells compared to MOCK control cells (BT-474 and SNU-251; Fig. 4G, H), further supporting that SOX5 overexpression inhibits DSBs.

This study further investigated how SOX5 suppresses DSBs. Because BRCA1/2 and RAD51 play key roles in HR repair upon DSBs [31], previous results from this study also indicated that olaparib-resistant cells overexpressed HR-associated genes, such as BRCA1 and RAD51 (Fig. 1D). Western blot analysis demonstrated that RAD51 and BRCA1 expression was upregulated in SOX5-overexpressed cells compared to MOCK control cells (Fig. 4I, J), suggesting that SOX5 drives the expression of HR-associated genes, RAD51 and BRCA1, which in turn repair DSBs, consequently developing PARP inhibitor resistance.

### SOX5 inhibition attenuates HR repair signaling via the Hippo-YAP pathway

**Fig. 5:**
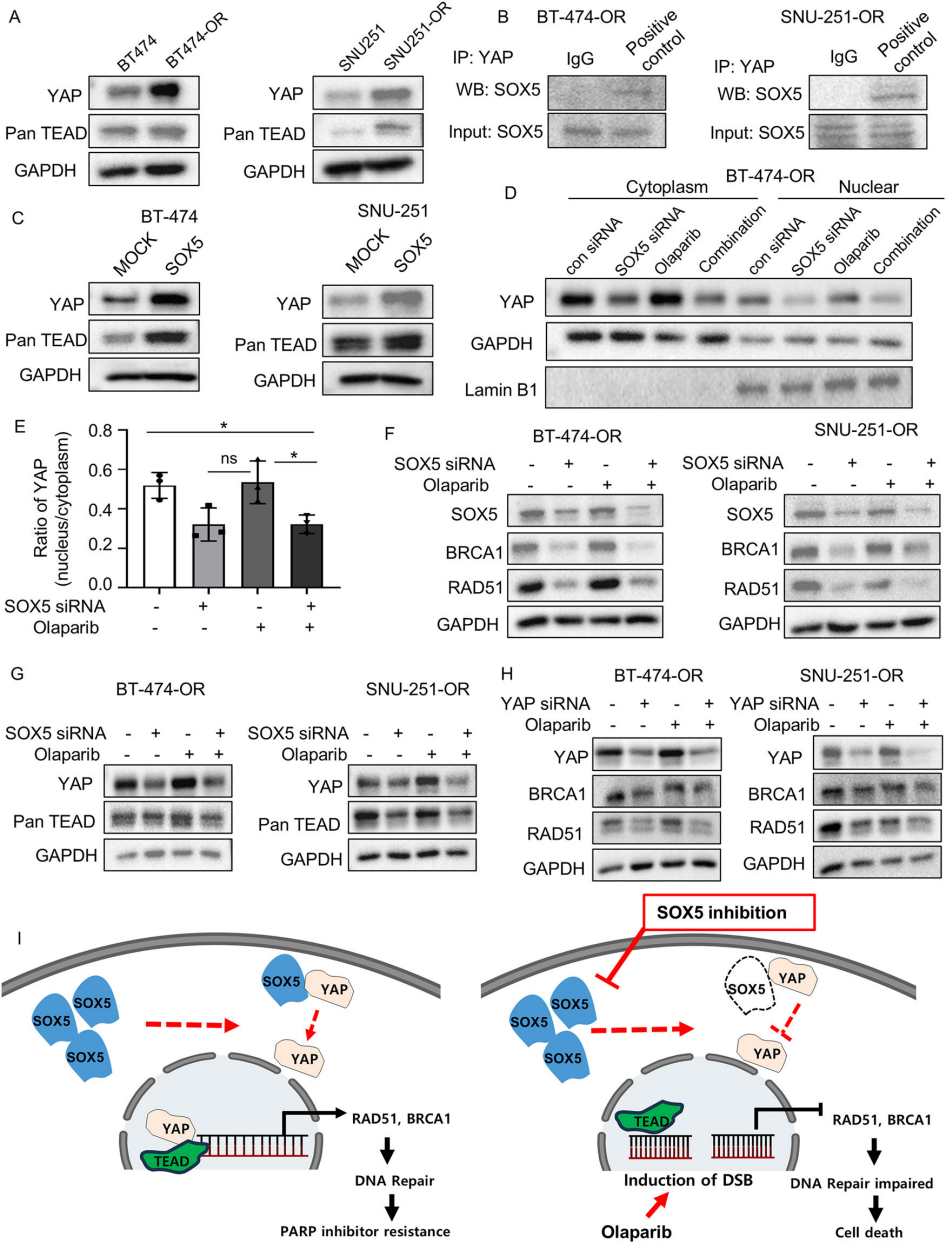
SOX5 inhibition attenuates HR repair signaling via the Hippo-YAP pathway. **A** Western blot analysis showed expression of YAP and pan-TEAD in parental versus olaparib-resistant cell. GAPDH was used as loading control. **B** The complex formation of YAP and SOX5 was analyzed by co-immunoprecipitation assay. **C** Western blot analysis showed expression of YAP and pan-TEAD in BT-474 and SNU-251 cells with and without ectopically overexpression of SOX5. GAPDH was used as loading control. **D**, **E** Western blot analysis showed YAP expression in the cytoplasm and nuclear fraction of BT-474-OR cells treated with SOX5 siRNA, olaparib, and their combination for 72 h. GAPDH and Lamin B1 was used as loading control. Bar graphs exhibited a ratio (nuclear/cytoplasmic) of YAP intensities from 3 independent experiments. Data are presented as mean ± SD. *P* values were calculated by Student’s *t*-test, indicating *p < 0.05, ns, not significant. **F** Western blot analysis showed expression of BRCA1 and RAD51 in olaparib-resistant cells treated with SOX5 siRNA, olaparib, and their combination for 72 h. GAPDH was used as loading control. **G** Western blot analysis showed the expression of YAP and pan-TEAD in olaparib-resistant cells after treatment with SOX5 siRNA, olaparib, and their combination for 72 h. GAPDH was used as loading control. **H** Western blot analysis showed the expression of BRCA1 and RAD51 in olaparib-resistant cells after treatment with YAP siRNA, olaparib and their combination for 72 h. GAPDH was used as loading control. **I** Schematic diagram showing the proposed mechanism that explains how SOX5 is associated with PARP inhibitor resistance. Briefly (left panel), in the olaparib-resistant cells SOX5 is upregulated in the olaparib-resistant cells, where SOX5 interacts with YAP. Then YAP is translocated into the nucleus, transcribing HR repair genes (BRCA1 and RAD51) and consequently PARP inhibitor resistance occurs. On the contrary, (right panel) when SOX5 is inhibited in the presence of PARP inhibitors, nuclear translocation of YAP is suppressed. Subsequently, transcription of HR repair genes (BRCA1 and RAD51) is attenuated, resulting in profound DNA DSBs and cell death.

Based on a previous study, SOX5 interacts with yes-associated protein 1 (YAP1) [20], which is a transcriptional coactivator with the PDZ-binding motif (TAZ), the major downstream effector of the Hippo pathway [32]. The main partners of YAP/TAZ are TEA domain family members (TEAD1–TEAD4) [33]. Therefore, to determine whether SOX5 interacts with YAP, this study first evaluated YAP and pan-TEAD expression in parental cells (BT-474 and SNU-251) versus olaparib-resistant cells (BT-474-OR and SNU-251-OR), showing YAP and pan-TEAD upregulation in olaparib-resistant cells compared to their corresponding parental cells (Fig. 5A). The coimmunoprecipitation assay confirmed the complex formation between SOX5 and YAP in BT-474-OR and SNU-251-OR cells (Fig. 5B and Supplementary Fig. S4A, B). Moreover, YAP and pan-TEAD expression was upregulated in ectopically SOX5-overexpressed cells (Fig. 5C), suggesting that SOX5 induced YAP expression.

Based on a previous study, if the Hippo pathway is off, YAP is translocated into the nucleus and functions as a transcriptional coactivators to induce cell proliferation and anti-apoptosis [34]. We observed that nuclear translocation of YAP was promoted in olaparib-resistant cells (BT-474-OR and SNU-251-OR) after the treatment of olaparib compared to SOX5 siRNA and combination treatment as if the Hippo pathway was off. In contrast, SOX5 knockdown alone or in combination with olaparib dramatically suppressed the nuclear translocation of YAP, as if the Hippo pathway turned on again (Fig. 5D, E; Supplementary Fig. S4C, D). We further examined YAP phosphorylation at serine 127 (S127) to assess its nuclear translocation. Phosphorylation at S127 promotes cytoplasmic sequestration, preventing nuclear localization, whereas non-phosphorylated YAP at this site typically indicates nuclear translocation and active transcriptional function [35]. Treatment with SOX5 siRNA, both alone and in combination, increased p-YAP (S127) levels, suggesting cytoplasmic retention, whereas olaparib treatment reduced p-YAP (S127) expression, indicating nuclear translocation (Supplementary Fig. S4E, F). To further investigate YAP1 accumulation in the nucleus, we performed immunofluorescence after overexpressing SOX5 and EGFP-YAP1 in parental cells, as well as EGFP-YAP1 in resistant cells. Nuclear translocation of YAP1 was observed in SOX5-overexpressing parental cells but not in control parental cells. Additionally, nuclear translocation of YAP1 was also detected in resistant cells, likely due to the high expression of SOX5 in these cells (Supplementary Fig. S4G–J). Collectively, these data indicate that SOX5 interacts with YAP, translocating YAP into the nucleus by suppressing the Hippo pathway, where YAP serves as a transcriptional coactivator.

Because YAP/TAZ-TEAD transcriptional activity governs RAD51 expression in cancer cells [36], whether SOX5 inhibition regulates YAP and the HR repair genes, BRCA1 and RAD51, in olaparib-resistant cells was investigated. SOX5 siRNA alone or the combination of SOX5 siRNA and olaparib (or talazoparib) dramatically inhibited BRCA1 and RAD51 expression (Fig. 5F; Supplementary Fig. S5A, B). To clarify the mechanism of how SOX5 inhibition attenuates BRCA1 and RAD51 expression, YAP and pan-TEAD expression in olaparib-resistant cells (BT-474-OR, SNU-251-OR) after treatment with SOX5 siRNA, olaparib (or talazoparib), or combination was analyzed, showing YAP and pan-TEAD suppression in SOX5 siRNA or combination-treated cells (Fig. 5G; Supplementary Fig. S5C, D). To further validate whether YAP influences BRCA1 and RAD51 expression, YAP was knocked down using siRNA, and YAP knockdown alone or in combination with olaparib (or talazoparib) considerably suppressed BRAC1 and RAD51 expression (Fig. 5H; Supplementary Fig. S5E, F). Together, these data proved that SOX5 inhibition inactivated YAP and subsequently suppressed nuclear translocation, finally losing its transcriptional activity of the target genes, BRCA1 and RAD51 (Fig. 5I). Therefore, SOX5 inhibition in the presence of PARP inhibitors attenuated HR repair genes, RAD51 and BRCA1, via YAP inactivation, suggesting that SOX5 inhibition in combination with PARP inhibitor may overcome PARP inhibitor resistance by suppressing the HR repair pathway.

### Combined SOX5 siRNA and olaparib regresses olaparib-resistant breast cancer cells synergistically in a xenograft model

**Fig. 6:**
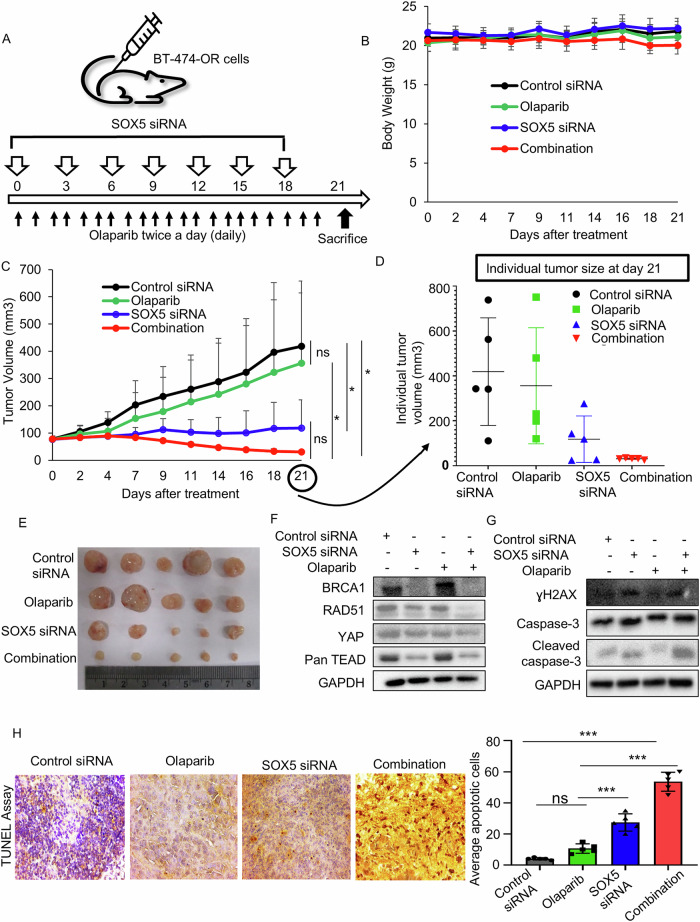
Combined SOX5 siRNA and olaparib regresses the olaparib-resistant breast cancer synergistically in a xenograft model. **A** Schematic view of in vivo efficacy experiment in acquired olaparib-resistant xenograft model using BT-474-OR cells. When tumor reached 70–80 mm^3^, mice were randomized to control siRNA (*n* = 5), olaparib (*n* = 5), SOX5 siRNA (*n* = 5) and combination (*n* = 5) treatment groups. Control siRNA and SOX5 siRNA were administered intratumorally every 3 days for 7 times. Olaparib was administered by oral gavage twice daily for 21 days. **B** Average body weight of each treatment group. Error bars represent the SD of 5 mice per group. **C** Mean tumor growth inhibition curves in mice treated with indicated drugs. Error bars represent the SD of 5 mice per group. Indicated *p* values were calculated by Student’s *t*-test, indicating *p < 0.05. ns, not significant. **D** Individual tumor volumes were shown on day 21 of treatment initiation. Data were presented as the mean ± SD. **E** Photo of the excised tumors of each treatment group shown after sacrificing on day21. **F** Western blot using xenograft tumors after 21 days of treatment initiation. Combination treatment suppressed the expressions of BRCA1, RAD51, YAP and pan-TEAD more than did olaparib alone. **G** Western blot using xenograft tumors after 21 days of treatment initiation. The expressions of DNA damage marker (ɣH2AX) and apoptosis marker (cleaved caspase-3) were more increased after combination treatment than monotherapy. **H** TUNEL assay using xenograft tumors. Staining images of each group are shown, and bar graph represented average apoptotic cells in each group in five random, non-overlapping fields at 400x magnification. Data are presented as mean ± SD. Indicated *p* values were calculated by Student’s *t*-test, indicating ***p < 0.001. ns, not significant.

Based on enhanced PARP inhibitor sensitivity by SOX5 inhibition in vitro, whether the addition of SOX5 siRNA could promote the PARP inhibitor (olaparib) response in vivo was investigated. The olaparib-resistant breast cancer xenograft mouse model was established using BT-474-OR cells. When the tumor reached 70–80 mm^3^, control siRNA and SOX5 siRNA were administered intratumorally every 3 days, and olaparib was orally administered twice daily for 3 weeks. Mice were sacrificed on day 21 of drug treatment (Fig. 6A). SOX5 siRNA and the combination of SOX5 siRNA and olaparib were well tolerated, and no generalized toxicity was observed as the body weight remained normal (Fig. 6B). Significant tumor growth inhibition was observed after combining SOX5 siRNA with olaparib compared to olaparib alone (*p* < 0.05) and control siRNA (*p* < 0.05). Noticeably, SOX5 siRNA alone significantly regressed tumor growth than control siRNA (*p* < 0.05), but the combination group exhibited a greater degree of tumor regression compared to SOX5 siRNA alone, paralleling in vitro results. However, olaparib treatment did not inhibit tumor growth compared to the nontreatment control (i.e., control siRNA), indicating that the xenograft model was olaparib-resistant (Fig. 6C). Graphs of individual tumor volumes on day 21 showed substantial variations of tumor volumes within each treatment group. However, profound tumor regression was observed without variation in the combination treatment group (Fig. 6D). Photographs of the excised tumors are shown in Fig. 6E. Western blot analysis of xenograft tumors showed noticeable suppression of HR proteins (i.e., BRCA1 and RAD51) and YAP, pan-TEAD in the combination group compared to olaparib alone and control siRNA (Fig. 6F). The expression of ɣH2AX and cleaved caspase-3 was noticeably elevated in the combination treatment, suggesting an increase of DSB-induced apoptosis (Fig. 6G). TUNEL assay using xenograft tissues indicated that SOX5 siRNA increased apoptosis compared to control siRNA (*p* < 0.001), and the combination treatment increased apoptosis more remarkably than SOX5 siRNA monotherapy (*p* < 0.001; Fig. 6H).

### High SOX5 expression is associated with poor prognosis in ovarian cancer

**Fig. 7:**
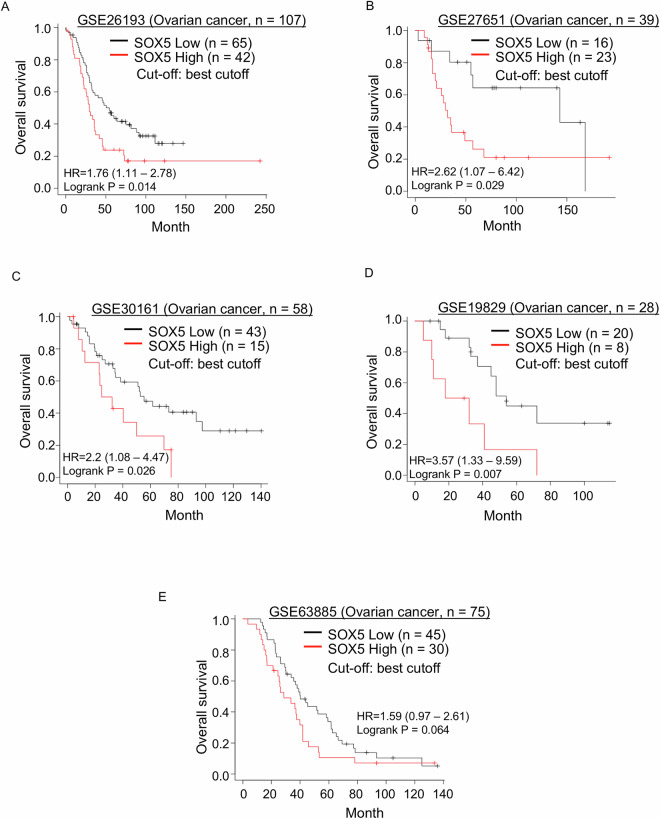
High SOX5 expression is associated with poor prognosis in ovarian cancer. **A**–**E** Kaplan–Meier survival curve of OS in ovarian cancer according to relative SOX5 mRNA expression, and the data were analyzed using online platform Kaplan–Meier plotter (https://kmplot.com/analysis/). SOX5 mRNA expression levels were divided into two groups, “SOX5-High” and “SOX5-Low,” with the auto-select best cutoff.

Independent public mRNA expression data sets were used to evaluate the impact of SOX5 overexpression on ovarian cancer prognosis. As described in the Materials and Methods, to determine an optimal cutoff point for SOX5-High and SOX5-Low groups, the minimum *p* value approach was used in predicting OS [29]. The cutoffs were set at the auto-select best cutoff for all data sets. The SOX5-High group exhibited shorter OS than the SOX5-Low group in five of seven data sets of ovarian cancer patients (Fig. 7A–E), although GSE18520 and GSE9891 analyses did not support our data (data not shown). Altogether, based on these public mRNA databases, this significant association between SOX5 expression and poor prognosis supported the clinical relevance of SOX5 as a therapeutic target.

## Discussion

The eventual progression of BRCA1/2-mutated breast or ovarian cancer, even after treatment with PARP inhibitors, has limited the success of this therapy in the clinic, emphasizing the need to identify mechanisms of this acquired resistance. To explore the mechanisms of PARP inhibitor resistance and overcoming strategies, we established reliable olaparib-resistant breast and ovarian cancer preclinical models, as demonstrated by the 4.5-fold increase in the IC_50_ of the drug. Our preclinical models confirmed that increased activity of HRR genes, BRCA1/2 or RAD51, is associated with acquired PARP inhibitor resistance, as published in previous studies [37]. This study demonstrated that SOX5 overexpression accounted for PARP inhibitor resistance. From the therapeutic perspective, SOX5 inhibition, in combination with olaparib, could overcome PARP inhibitor resistance. Considering the wide use of PARP inhibitors in BRCA1/2-mutated cancers, we suggest that our resistant model should be very valuable in developing drugs to overcome resistance.

This mechanistic study suggested that SOX5 drives PARP inhibitor resistance by enhancing the HRR capacity via the Hippo-YAP/TAZ pathway. Mechanistically, in more detail, upregulated SOX5 activates and translocases YAP into the nucleus by suppressing the Hippo pathway. In the nucleus, YAP functions as a transcriptional coactivator with the help of TEAD and augments the expression of HRR genes such as BRCA1 and RDA51. As a result, DSB repair is promoted, and PARP inhibitor resistance occurs. Previous studies also suggested YAP-induced upregulation of the HRR capacity by enhancing HR pathway genes, supporting our data [36]. In recent years, the Hippo-YAP/TAZ signaling pathway has gained enormous attention due to its role in various anticancer drug resistance [38]. Furthermore, several preclinical studies demonstrated that YAP/TAZ was involved in resistance to DNA-damaging drugs (e.g., cisplatin, temozolomide, doxorubicin, and etoposide) [38]. Despite an association of YAP/TAZ and resistance to various DNA-damaging drugs reported previously, the role of YAP/TAZ in PARP inhibitor resistance has never been reported yet. Hence, this study suggested an activated SOX5-YAP/TAZ pathway as a novel mechanism accounting for PARP inhibitor resistance. Combined SOX5 inhibition and olaparib could overcome PARP inhibitor resistance by suppressing the HRR capacity. Mechanistically, SOX5 inhibition suppresses YAP and inactivates HRR genes, such as BRCA1 and RAD51, consequently increasing DNA DSBs and apoptosis of PARP inhibitor-resistant cells.

In addition to the SOX5-YAP/TAZ pathway, preclinical studies reported that restoration of the HRR capacity via the activation of various survival pathways contributes to PARP inhibitor resistance. For instance, Wnt/β-catenin, transforming growth factor-β, vascular endothelial growth factor (VEGF) receptor, RAS, phosphatidylinositol 3-kinase (PI3K)-AKT, and AR signaling pathways promoted HRR activity and PARP inhibitor resistance in previous studies [18, 39, 40]. There are no direct inhibitors of HR pathway. An alternative approach to suppress the restoration of HR in patients receiving PARP inhibitors might be the use of inhibitors targeting the aforementioned survival pathways. These survival pathway inhibitors interfere with gene expression, nuclear localization, or the recruitment of HR proteins, ultimately functioning as an indirect HR inhibitor [41]. Inhibition of these survival pathways and the DDR pathway showed the potential to pharmacologically induce HRD-equivalent conditions and lead to synthetic lethality with PARP inhibitor [18]. Several clinical trials supported this notion, such that combining VEGF antagonist and olaparib or niraparib improved PFS in ovarian cancer patients [42, 43]. Furthermore, a phase Ib clinical trial is evaluating the combination of niraparib and copanlisib, a PI3K inhibitor, in BRCA1/2-mutated ovarian, endometrial, or fallopian tube cancer (NCT03586661). Other phase Ib/II clinical trials are evaluating the efficacy of olaparib in combination with capivasertive (AKT inhibitor) or vistusertib (mTORC1/2 inhibitor) in BRCA1/2-mutated recurrent endometrial and ovarian cancer (NCT02208375). As mentioned above, the activity of these inhibitors might be an indirect inhibition of HR through regulating the DDR pathway genes, such as BRCA1, RAD51, wee1 G2 checkpoint kinase (WEE1), and DNA topoisomerase 2-binding protein 1 (TOPBP1) [41]. In accordance with this, our data suggested that SOX5 inhibition indirectly suppresses the expression of the HRR protein, BRCA1, and RAD51 by inactivating YAP. Therefore, SOX5 inhibitors, in combination with PARP inhibitors, could be a potential strategy to inhibit HRR indirectly and overcome PARP inhibitor resistance. Based on these scientific rationales and the fact that there are no available SOX5 inhibitors, it is worth developing SOX5 inhibitors to overcome PARP inhibitor resistance.

To speculate the potential toxicities of SOX5 inhibition, we attempted to search for studies regarding consequences in SOX5-knockout mice. In a relevant study, SOX5 single-null mice were born with mild skeletal abnormalities, and SOX5 double-null embryos died on day 16.5 of gestation due to severe, generalized chondrodysplasia [44]. These results indicated that SOX5 is necessary for embryonic development and postnatal growth and development. Therefore, in clinics, careful attention is needed while administering SOX5 inhibitors to a patient with child-bearing potential. Nevertheless, no weight loss was observed in the in vivo study, indicating that SOX5 inhibition had no generalized toxicities to adult tissues in the mouse model. In addition, reported common toxicities of PARP inhibitors are nausea, vomiting, bone marrow suppression, and the development of hematologic malignancies [45]. However, further studies are required to better understand overlapped toxicities of the combined SOX5 and PARP inhibitors if they are given together.

In conclusion, this study demonstrated that SOX5 overexpression is a novel mechanism behind PARP inhibitor resistance by repairing DSBs through HRR. Our study also validated SOX5 as a promising therapeutic target by demonstrating that SOX5 inhibition in combination with PARP inhibitor shows synergistic anticancer activity in PARP inhibitor-resistant preclinical cancer models. There are no SOX5 inhibiting drugs available to date. Therefore, developing an SOX5 inhibitor is a rational approach to overcome PARP inhibitor resistance.

## Supplementary information

Supplementary figures and legends

Supplementary Table

SOX5_Uncropped western blot

## Data Availability

The data that support the findings of this study are available from the corresponding author upon reasonable request.
