# Supplementary material for: SOX5 inhibition overcomes PARP inhibitor resistance in BRCA-mutated breast and ovarian cancer
Source: Cell Death Dis. 2025 Apr 24;16(1):333. doi: 10.1038/s41419-025-07660-7 (PMC12022250; doi:10.1038/s41419-025-07660-7)
Supplement: Supplementary file 1 — Supplementary figures and legends [file 41419_2025_7660_MOESM1_ESM.docx]

**SOX5 inhibition overcomes PARP inhibitor resistance in BRCA-mutated breast and ovarian cancer**

Mithun Ghosh ^1^, Min Sil Kang^1^, Nar Bahadur Katuwal^1^, Sa Deok Hong^1^, Seong Min Park^1^, Seul-Gi Kim^2^, Seung Ryeol Lee^3^, Yong Wha Moon^2^

^1^Department of Biomedical Science, The Graduate School, CHA University, Seongnam-si, Republic of Korea (13488)

^2^Hematology and Oncology, Department of Internal Medicine, CHA Bundang Medical Center, CHA University, Seongnam-si, Republic of Korea (13496)

^3^Department of Urology, CHA Bundang Medical Center, CHA University, Seongnam-si, Republic of Korea (13496)

**Address correspondence to:** Yong Wha Moon, Hematology and Oncology, Department of Internal Medicine, CHA Bundang Medical Center, CHA University, 59 Yatap-ro, Bundang-gu, Seongnam-si, Gyeonggi-do, Republic of Korea (13496), Tel: +82-31-780-3436, Fax: +82-31-780-3929, E-mail: [ymoon@cha.ac.kr](mailto:ymoon@cha.ac.kr)

**Supplementary figure & legends**


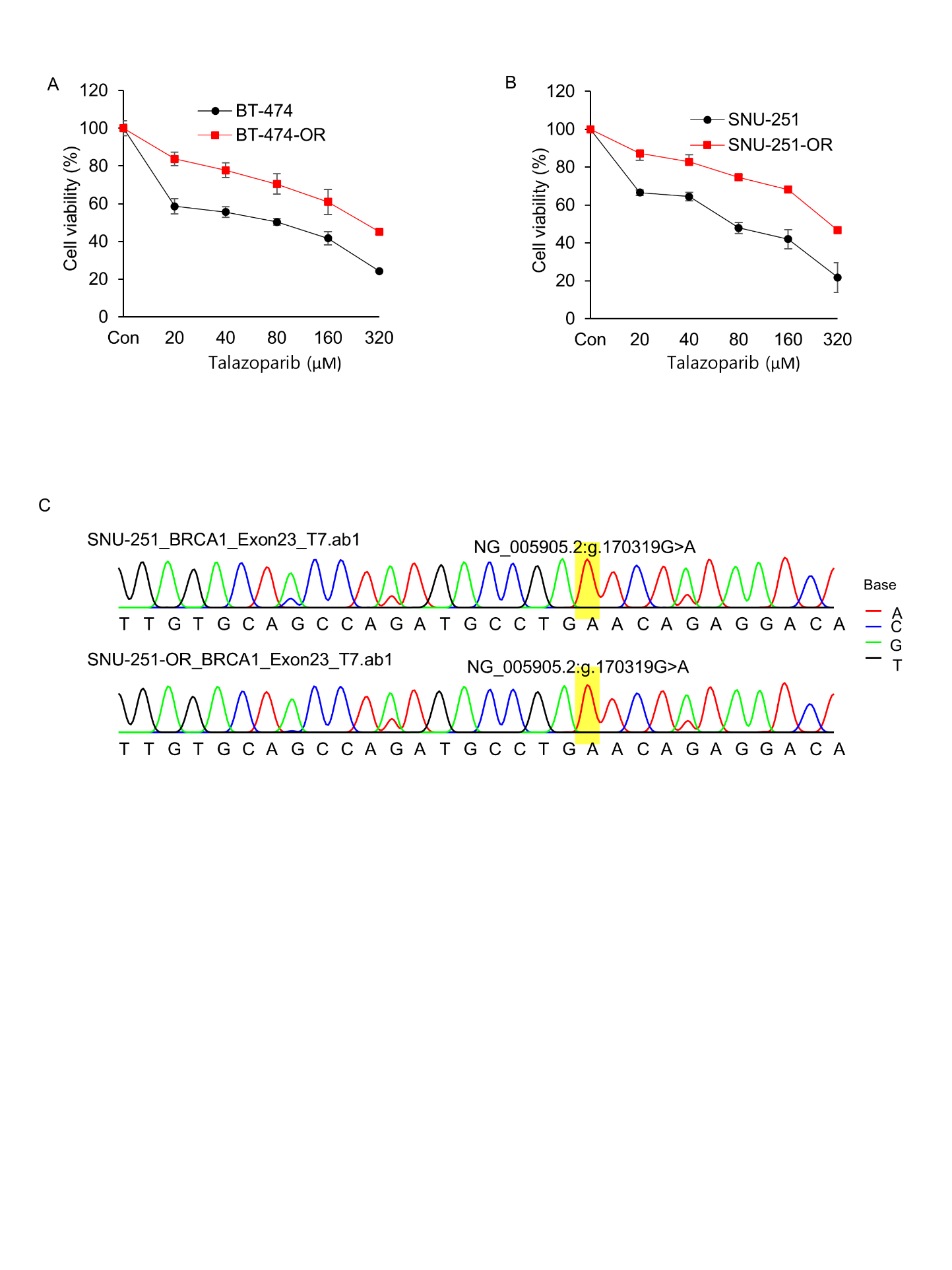


**Supplementary figure S1. Derivation and confirmation of olaparib-resistant cells**

**A**, **B** Olaparib-resistant cells were cross-resistant with another PARP inhibitor, talazoparib. Cell viability was measured by MTT assay. Cells were treated with various concentration talazoparib for 72 h. **C** Sequencing analysis of the BRCA1 gene in SNU-251 and SNU-251-OR cell lines. Partial sequences from SNU-251 (top) and SNU-251-OR (bottom) cell lines are displayed. The G-to-A base substitution at NG_005905.2:g.170319G>A in exon 23 is highlighted in yellow. The data indicate that SNU-251-OR cells retain the same mutation as the parental SNU-251 cells.


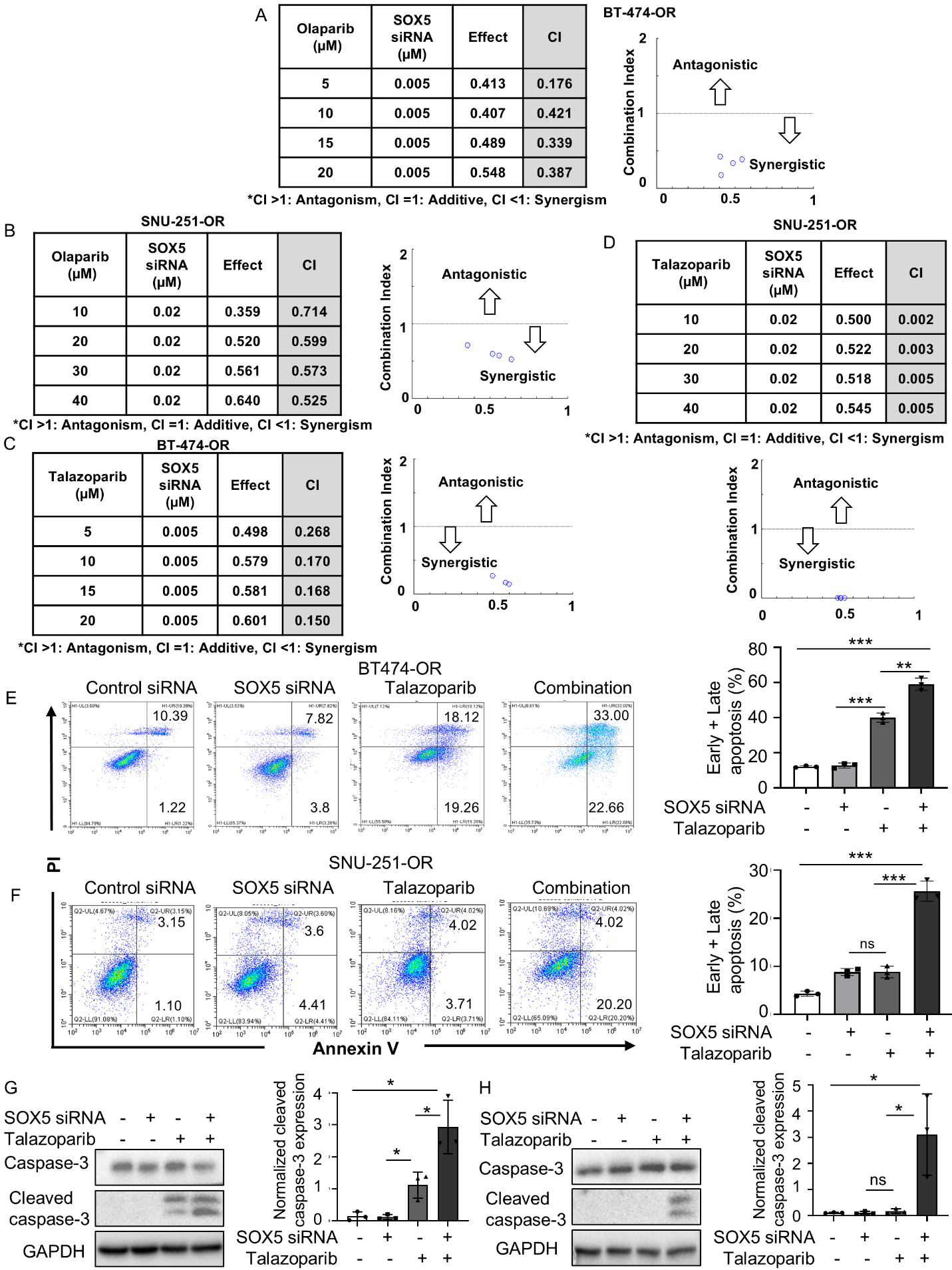


**Supplementary figure S2.** **SOX5 inhibition synergistically suppresses olaparib-resistant cells in combination with olaparib (or talazoparib) through DNA DSB**

**A**, **B** Combination Index (CI) value and CI plot of MTT assay in BT-474-OR and SNU-251-OR cells after treatment with SOX5 siRNA, olaparib and their combination for 72 h. **C**,**D** Combination Index (CI) value and CI plot of MTT assay in BT-474-OR and SNU-251-OR cells after treatment with SOX5 siRNA, talazoparib and their combination for 72 h. **E**,**F** Apoptosis assay conducted by flow cytometry. Representative scatter plots of PI (y-axis) and annexin V (x-axis) are shown. Cells were treated with SOX5 siRNA (5 nmol in BT474-OR and 20 nmol in SNU-251-OR), talazoparib (10 µM) and their combination for 72 h. The bar graphs depicted the average of total apoptotic cells of 3 independent experiments. *P*-values were calculated by Student’s *t*-test, indicating ***p* < 0.01, ****p* < 0.001. Data are presented as mean ± SD from 3 independent experiments. Abbreviation: ns, not significant **G**, **H** Western blot analysis showed the expressions of apoptosis marker, caspase-3, and cleaved caspase-3 in olaparib-resistant cells (BT-474-OR, SNU-251-OR). After treatment with SOX5 siRNA (5 nmol in BT474-OR and 20 nmol in SNU-251-OR) and talazoparib (5 µM) and their combination for 72 h. The bar graphs showed the normalized expression of cleaved caspase-3 based on densitometric analysis from 3 independent experiments. *P*-values were calculated by Student’s *t*-test, indicating **p* < 0.05. Abbreviation: ns, not significant. Data are presented as mean ± SD from 3 independent experiments. GAPDH was used as a loading control.


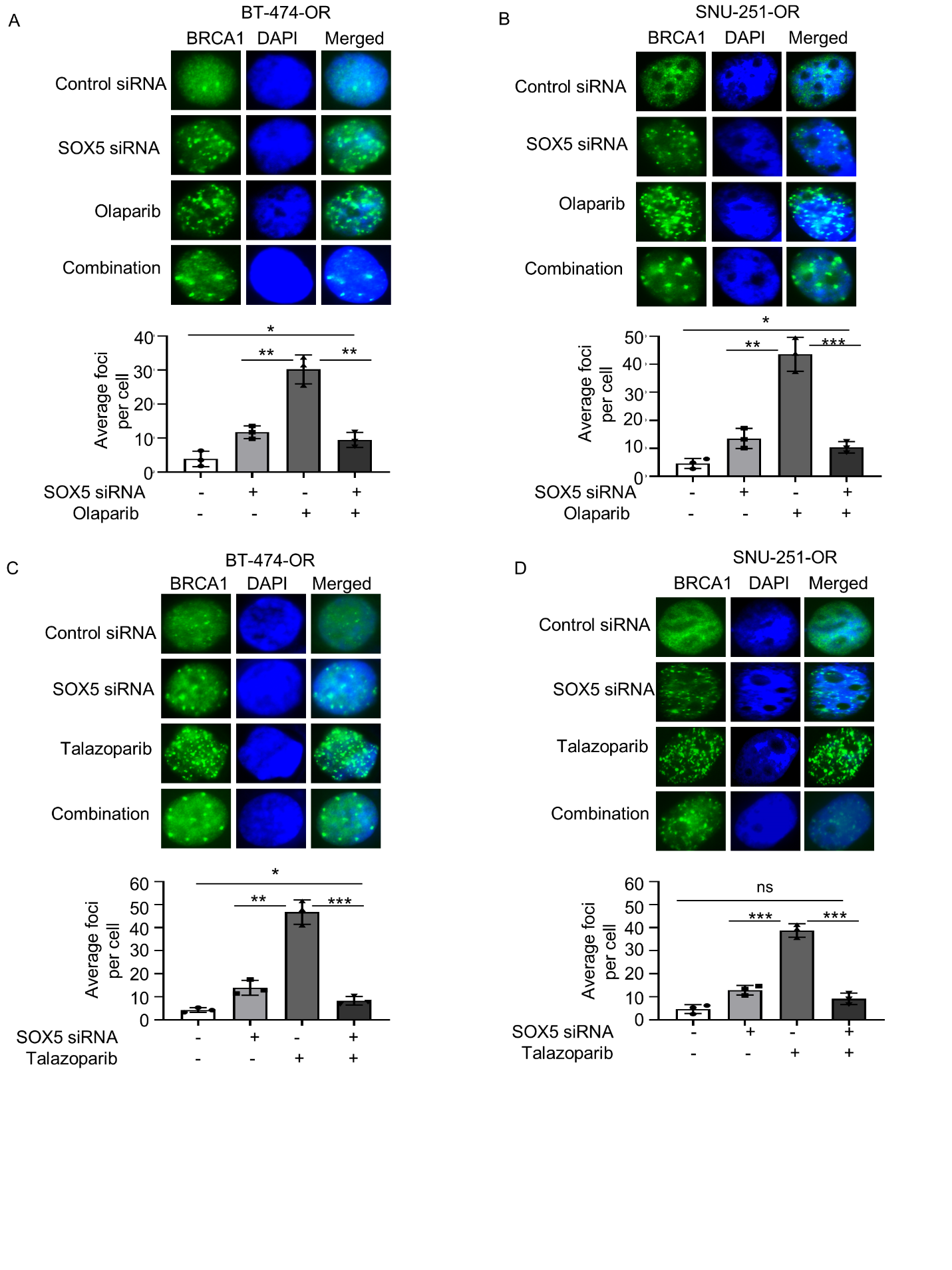


**Supplementary figure S3. SOX5 inhibition synergistically suppresses olaparib-resistant cells in combination with olaparib (or talazoparib) through DNA DSBA**

**A**-**D** Immunofluorescence images showed the BRCA1 foci formation in BT-474-OR and SNU-251-OR cells. Cells were treated with SOX5 siRNA (5 nmol in BT474-OR and 20 nmol in SNU-251-OR) and 5 µM of olaparib (or talazoparib, 5 µM) and their combination for 72 h**.** The images were taken at 100x magnification. The bar graphs depicted the average foci number per cell. Data were averaged from three independent experiments, with 100 cells analyzed per condition in each experiment. Data are presented as mean ± SD. *P*-values were calculated by Student’s *t*-test, indicating **p* < 0.05, ***p* < 0.01, ****p* < 0.001. Abbreviation: ns, not significant


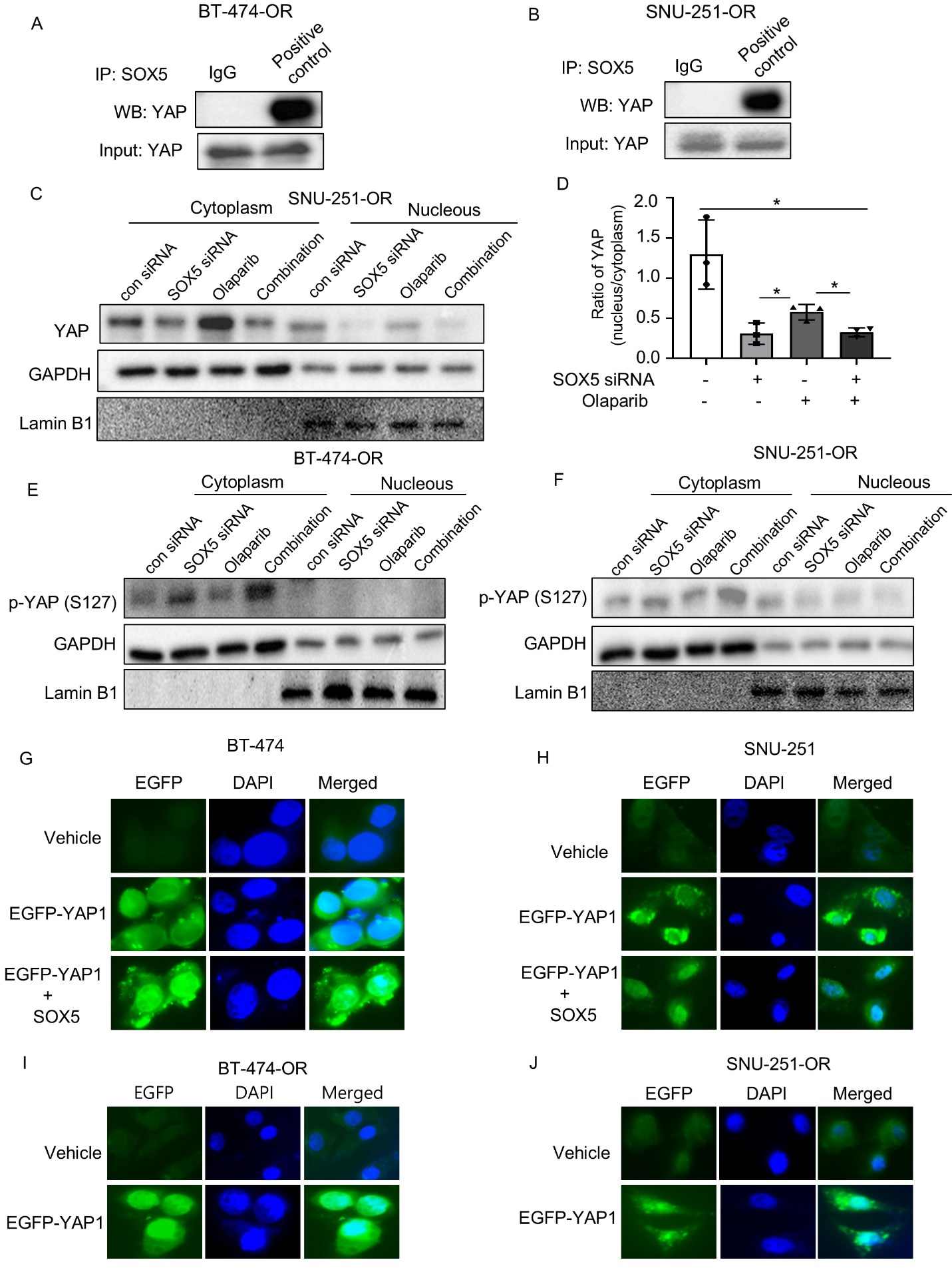


**Supplementary figure S4. SOX5 inhibition attenuates HR repair signaling via the Hippo-YAP pathway**

**A, B** The complex formation of YAP and SOX5 was analyzed by co-immunoprecipitation assay.

**C**, **D** Western blot analysis showed YAP expression in the cytoplasm and nuclear fraction of SNU-251-OR cells treated with SOX5 siRNA, olaparib and their combination for 72 h. GAPDH and Lamin B1 was used as loading control. Bar graphs exhibited a ratio (nuclear/cytoplasmic) of YAP intensities from 3 independent experiments. Data are presented as mean ± SD. *P*-values were calculated by Student’s *t*-test, indicating **p* < 0.05, abbreviation: ns, not significant. **E**, **F** Western blot analysis showed p-YAP (S127) expression in the cytoplasm and nuclear fraction of BT-474-OR and SNU-251-OR cells treated with SOX5 siRNA, olaparib and their combination for 72 h. GAPDH and Lamin B1 was used as loading control. **G-J** YAP1 nuclear translocation was determined by fluorescence microscope in parental (BT-474 and SNU-251) and resistant (BT-474-OR and SNU-251-OR) cells. Cells were transfected with EGFP-YAP1 alone, co-transfected with EGFP-YAP1 and SOX5, or treated with vehicle control.


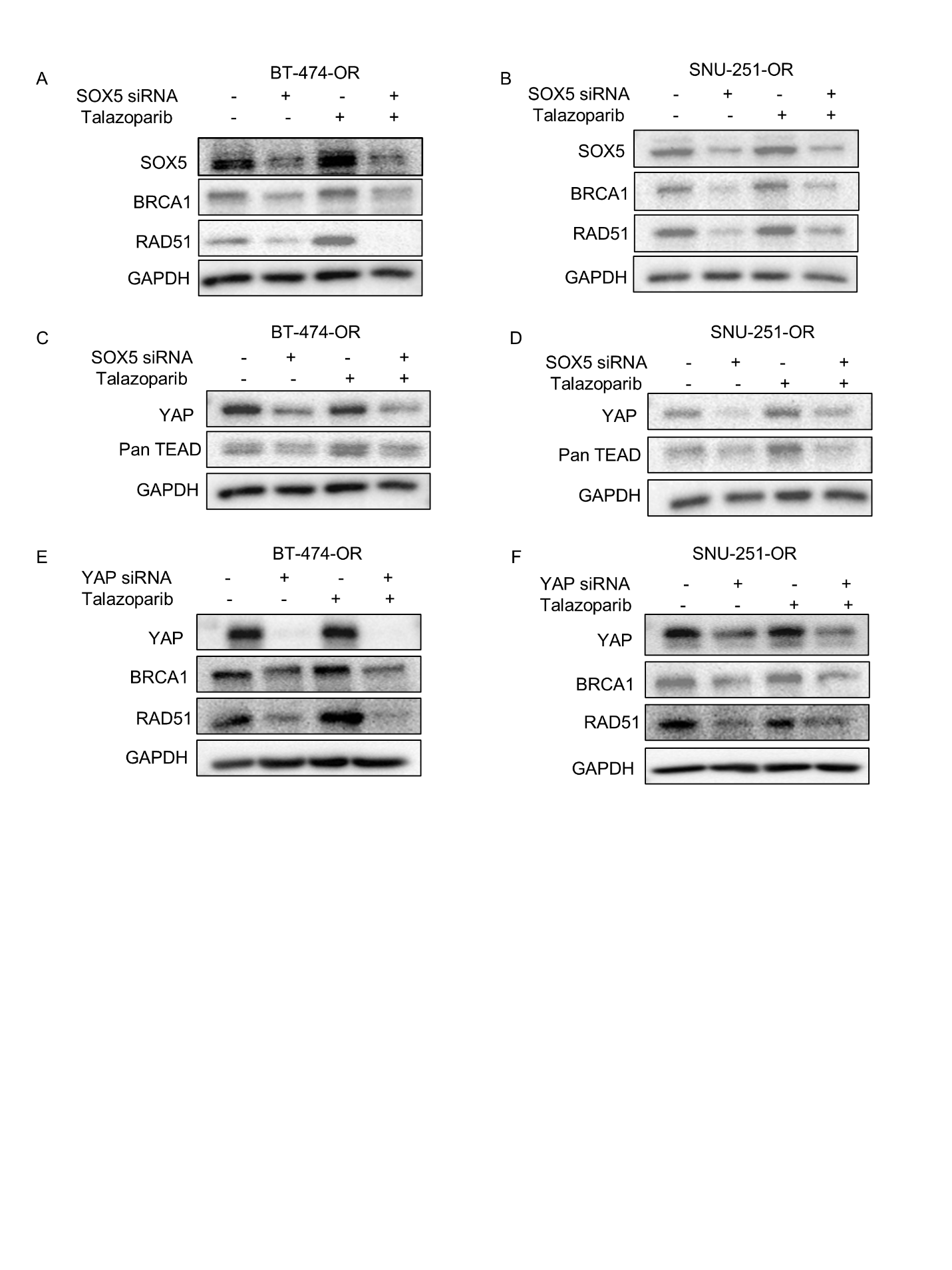


**Supplementary figure S5. SOX5 inhibition attenuates HR repair signaling via the Hippo-YAP pathway**

**A**, **B** Western blot analysis showed the expression of BRCA1 and RAD51 in olaparib-resistant cells (BT-474-OR, SNU-251-OR) treated with SOX5 siRNA, talazoparib and their combination for 72 h. GAPDH was used as loading control. **C**, **D** Western blot analysis showed the expression of YAP and pan-TEAD in olaparib-resistant cells (BT-474-OR, SNU-251-OR) after treatment with SOX5 siRNA, talazoparib and their combination for 72 h. GAPDH was used as loading control. **E**, **F** Western blot analysis showed the expression of BRCA1 and RAD51 in olaparib-resistant cells (BT-474-OR, SNU-251-OR) after treatment with YAP siRNA, talazoparib and their combination for 72 h. GAPDH was used as loading control.
