# Supplementary material for: SOX5 inhibition overcomes PARP inhibitor resistance in BRCA-mutated breast and ovarian cancer
Source: Cell Death Dis. 2025 Apr 24;16(1):333. doi: 10.1038/s41419-025-07660-7 (PMC12022250; doi:10.1038/s41419-025-07660-7)
Supplement: Supplementary file 2 — Supplementary Table [file 41419_2025_7660_MOESM2_ESM.docx]

**Supplemental Tables**

Supplemental Table S1. Information about histology, subtype, and BRCA1/2 mutation of BT-474 and SNU-251 cell.

| Cell | BT-474 | SNU-251 |
| --- | --- | --- |
| Cancer type | Breast | Ovarian |
| Histology | IDC (Invasive ductal carcinoma) | Endometroid carcinoma |
| Subtype | ER+HER2+ |  |
| BRCA mutation | BRCA2 mutated | BRCA1 mutated |
|  | 9281C > A; (S3094*) | Codon 1815 nonsense mutation, TGG(Trp) →TGA (stop) |
|  | Pathogenic | Pathogenic |

Supplemental Table S2. List of primary and secondary antibodies which were used in western blot and immunocytochemistry.

| Antibody | Host species | Dilution | Company (catalog#) | Application |
| --- | --- | --- | --- | --- |
| BRCA1 | Rabbit | 1:1000 | 14823, Cell signaling | WB |
| BRCA1 | Rabbit | 1:1000 | 50799, Cell signaling | ICC |
| RAD51 | Rabbit | 1:1000 | Ab133534, abcam | WB |
| SOX5 | Mouse | 1:500 | UM500047, Origene | WB |
| SOX5 | Rabbit | 1:50 | Ab94396, abcam | Co-IP |
| Caspase-3 | Rabbit | 1:1000 | 9662, Cell signaling | WB |
| ɣH2AX | RAbbit | 1:1000 | 5437, Cell signaling | WB, ICC |
| YAP | Rabbit | 1:1000, 1:50 | 14074, Cell signaling | WB, Co-IP |
| p-YAP1 (Ser127) | Rabbit | 1:1000 | MA5-33207, Invitrogen | WB |
| Pan-TEAD | Rabbit | 1:1000 | 13295, Cell signaling | WB |
| GAPDH | Rabbit | 1:1000 | 2118, Cell signaling | WB |
| Lamin B1 | Rabbit | 1:1000 | 13435S, Cell signaling | WB |
| Anti-Rabbit HRP | Goat | 1:5000 | GTX213110-01, Gene Tex | WB |
| Anti-Mouse HRP | Goat | 1:5000 | GTX213111-01, Gene Tex | WB |

Abbreviations: WB, western blot; ICC, immunocytochemistry

Supplemental Table S3. Primers used for qRT-PCR

| Primer name | Orientation | Sequence |
| --- | --- | --- |
| RAD51 | Forward | 5’- GAGAAGGTGTGGTGGTGTTATG -3’ |
|  | Reverse | 5’- ACTCACAGGGCAGTCTCTATAC -3’ |
| SOX5 | Forward | 5’- CCTGCCTGGTGGATGGCAAA -3’ |
|  | Reverse | 5’- GCTCTGGGCTGCTAGACACG -3’ |
| GAPDH | Forward | 5’- CAGCCTCAAGATCATCAGCA -3’ |
|  | Reverse | 5’- TGTGGTCATGAGTCCTTCCA -3’ |

Supplemental Table S4. A list of top 20 upregulated and downregulated genes in PARP inhibitor resistant cells.

| Gene Symbol | BT-474-OR/BT-474.fc |
| --- | --- |
| SCD5 | 64.73 |
| SNTB1 | 56.16 |
| PCDH9 | 55.77 |
| SOX5 | 46.24 |
| STEAP1 | 41.58 |
| LPAR3 | 41.25 |
| PRKG1 | 39.74 |
| IQGAP2 | 38.53 |
| COMMD3-BMI1 | 38.47 |
| TSPYL5 | 37.21 |
| GAD1 | 35.99 |
| HNF4G | 35.37 |
| LRRC8C | 35.30 |
| ARSJ | 33.81 |
| CHST1 | 32.54 |
| FAR2 | 30.67 |
| CPNE5 | 30.66 |
| RAI2 | 29.66 |
| HLA-DQB1 | 29.47 |
| ANKH | 28.77 |
| ANO5 | -30.1902 |
| PTGES3L-AARSD1 | -26.589 |
| ZNF22 | -24.9144 |
| CIDEB | -23.4594 |
| FMN2 | -17.6149 |
| LAMC2 | -15.1142 |
| LINC02210-CRHR1 | -12.8507 |
| SNX25P1 | -11.9156 |
| LOC101928725 | -10.7868 |
| ACTL10 | -10.4234 |
| ASPRV1 | -9.65812 |
| GREM2 | -9.49079 |
| TNFRSF6B | -9.48124 |
| RORB | -9.13673 |
| CRACR2A | -8.95183 |
| RAB39A | -8.93545 |
| CPNE8 | -8.77815 |
| ZNF512 | -8.452 |
| BMPER | -7.63483 |
| SH3TC1 | -7.25704 |
