# Supplementary material for: SOX5 inhibition overcomes PARP inhibitor resistance in BRCA-mutated breast and ovarian cancer
Source: Cell Death Dis. 2025 Apr 24;16(1):333. doi: 10.1038/s41419-025-07660-7 (PMC12022250; doi:10.1038/s41419-025-07660-7)
Supplement: Supplementary file 3 — SOX5_Uncropped western blot [file 41419_2025_7660_MOESM3_ESM.pdf]

Fig 1

D

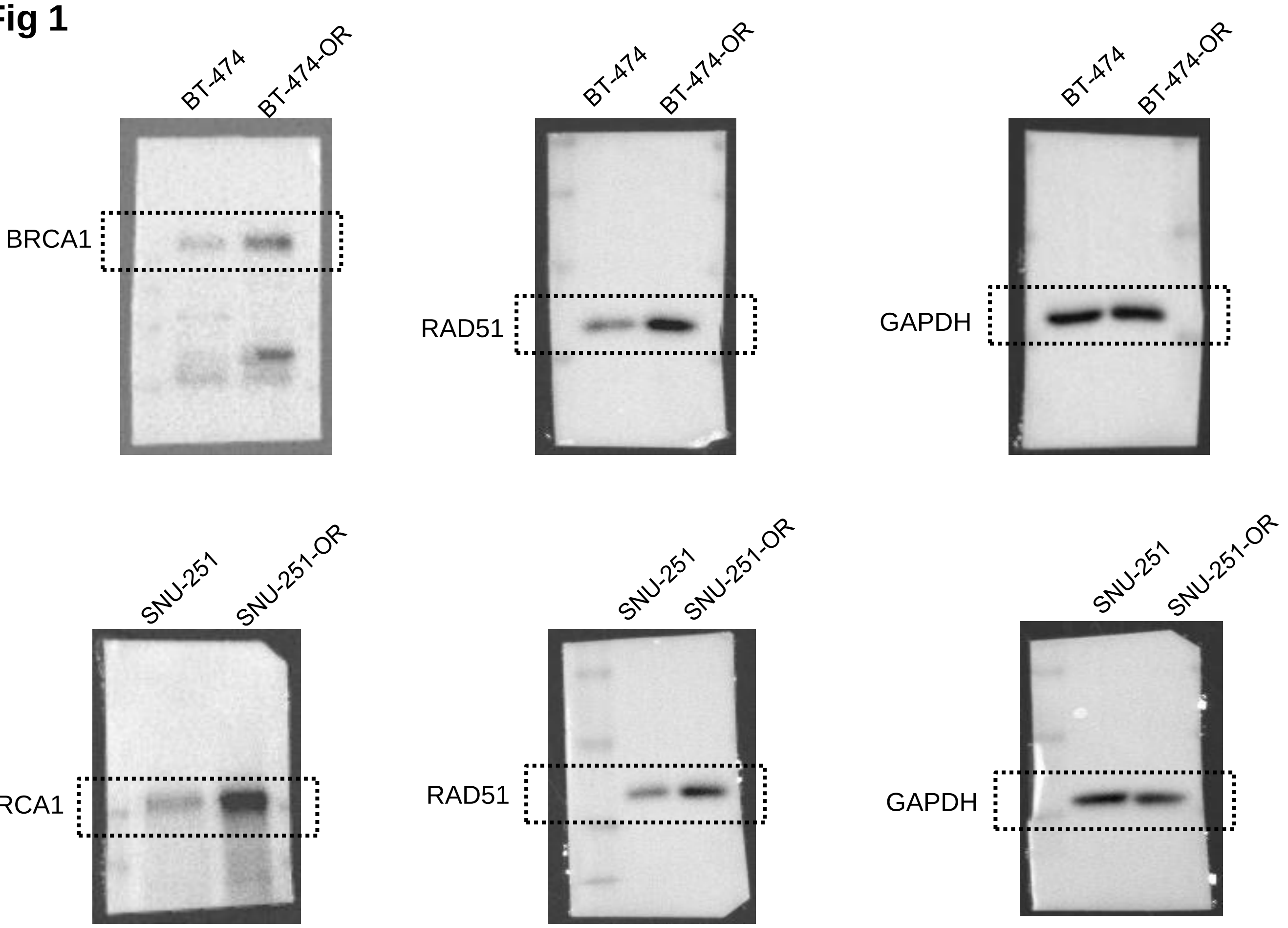

BT-474
BT-474-OR
BRCA1
RAD51
GAPDH
SNU-251
SNU-251-OR
BRCA1
RAD51
GAPDH

G

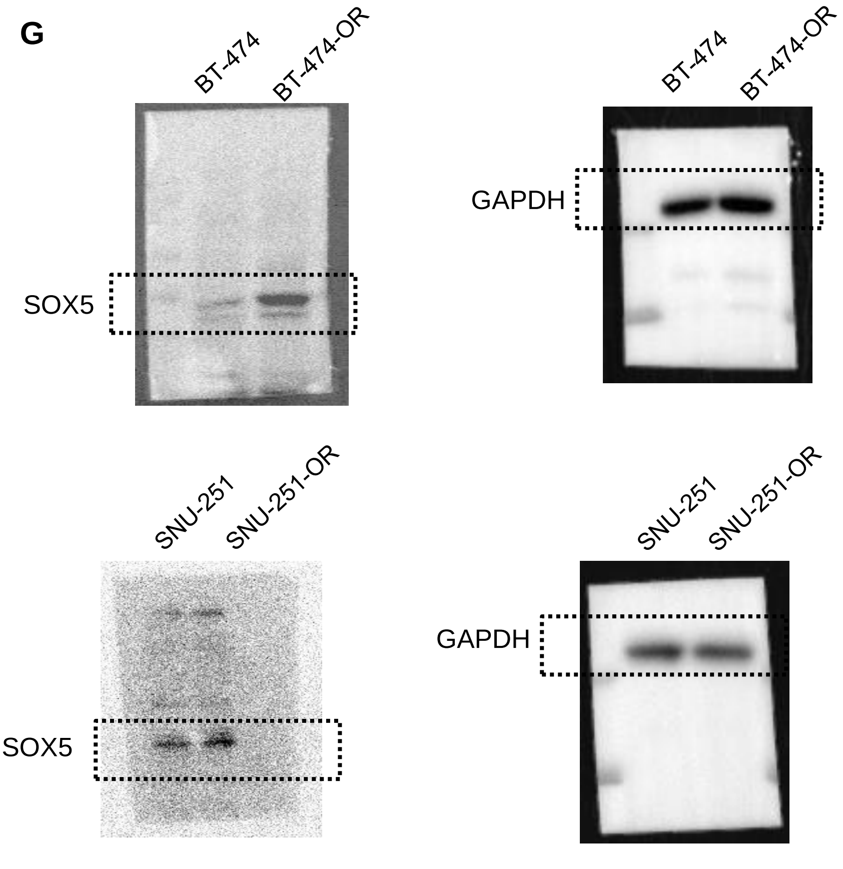

BT-474
BT-474-OR
SOX5
GAPDH
SNU-251
SNU-251-OR
SOX5
GAPDH

Fig 2

E

BT474-OR

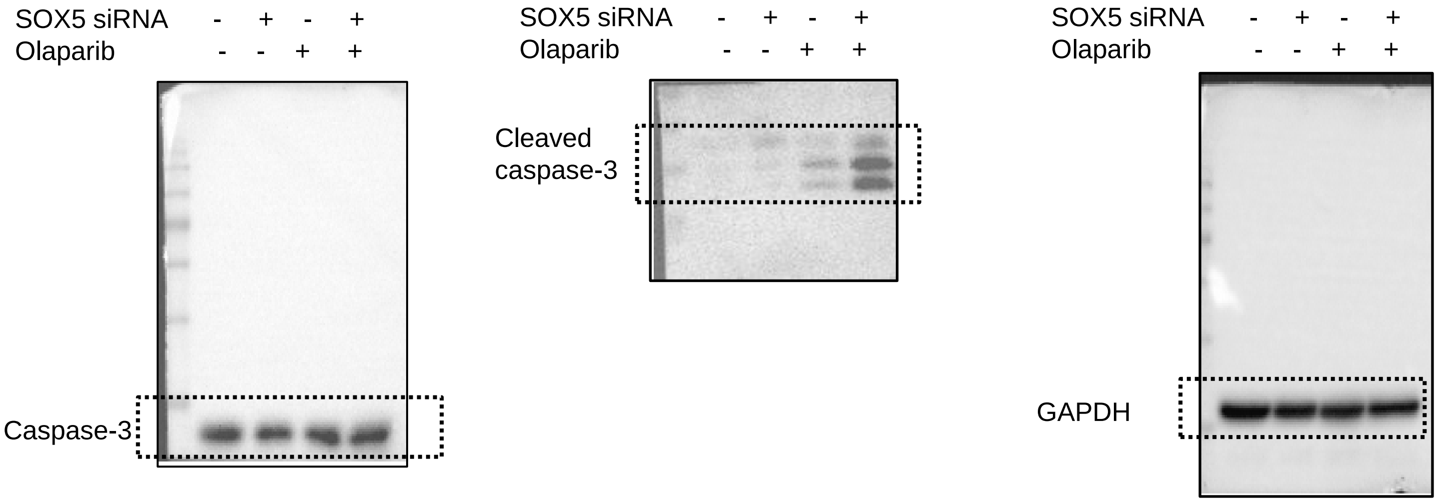

F

SNU-251-OR

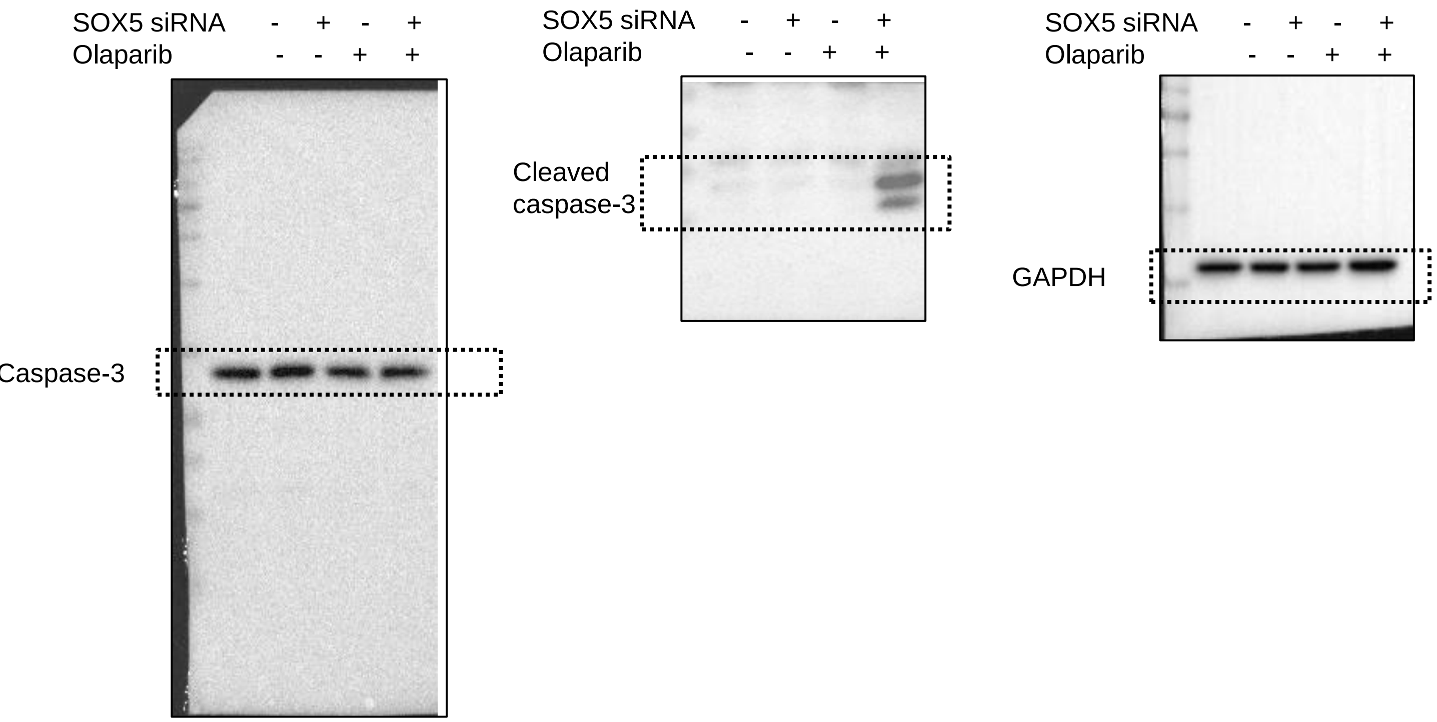

Fig 3

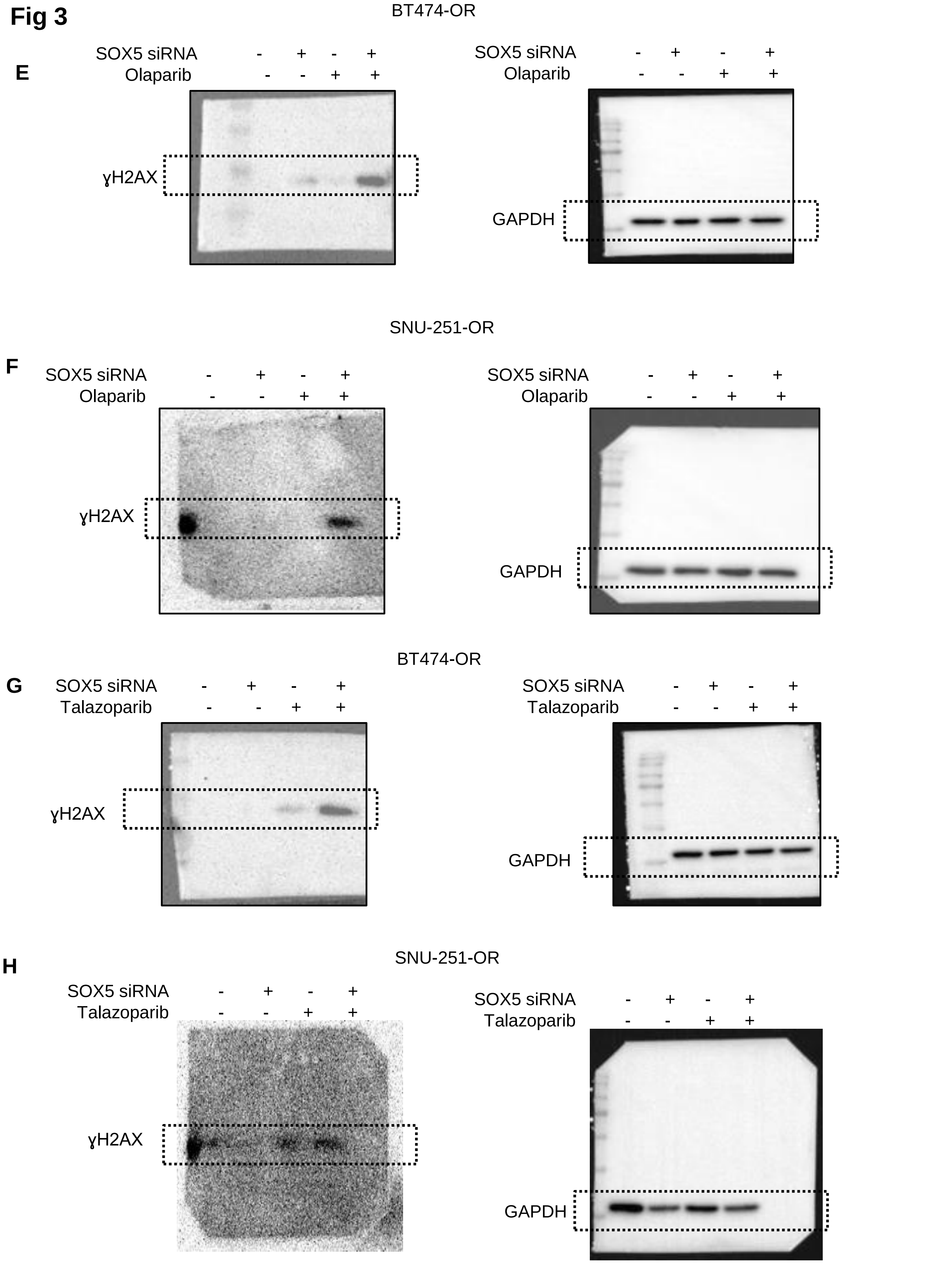
BT474-OR
E
SOX5 siRNA - + - +
Olaparib - - + +
ɣH2AX
SOX5 siRNA - + - +
Olaparib - - + +
GAPDH
SNU-251-OR
F
SOX5 siRNA - + - +
Olaparib - - + +
ɣH2AX
SOX5 siRNA - + - +
Olaparib - - + +
GAPDH
BT474-OR
G
SOX5 siRNA - + - +
Talazoparib - - + +
ɣH2AX
SOX5 siRNA - + - +
Talazoparib - - + +
GAPDH
SNU-251-OR
H
SOX5 siRNA - + - +
Talazoparib - - + +
ɣH2AX
SOX5 siRNA - + - +
Talazoparib - - + +
GAPDH

Fig 4

BT-474

G

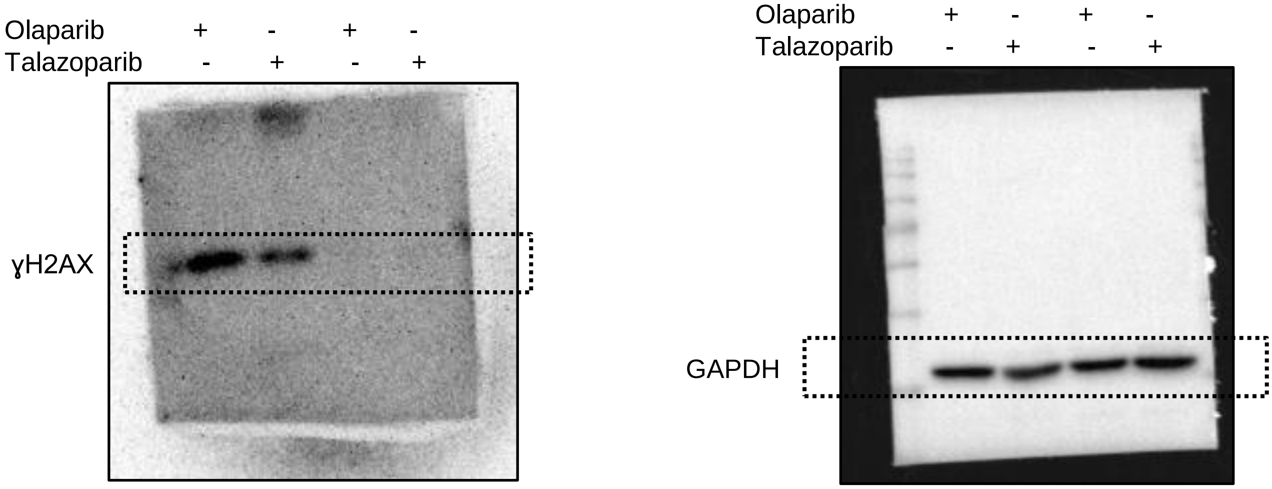

H

SNU251

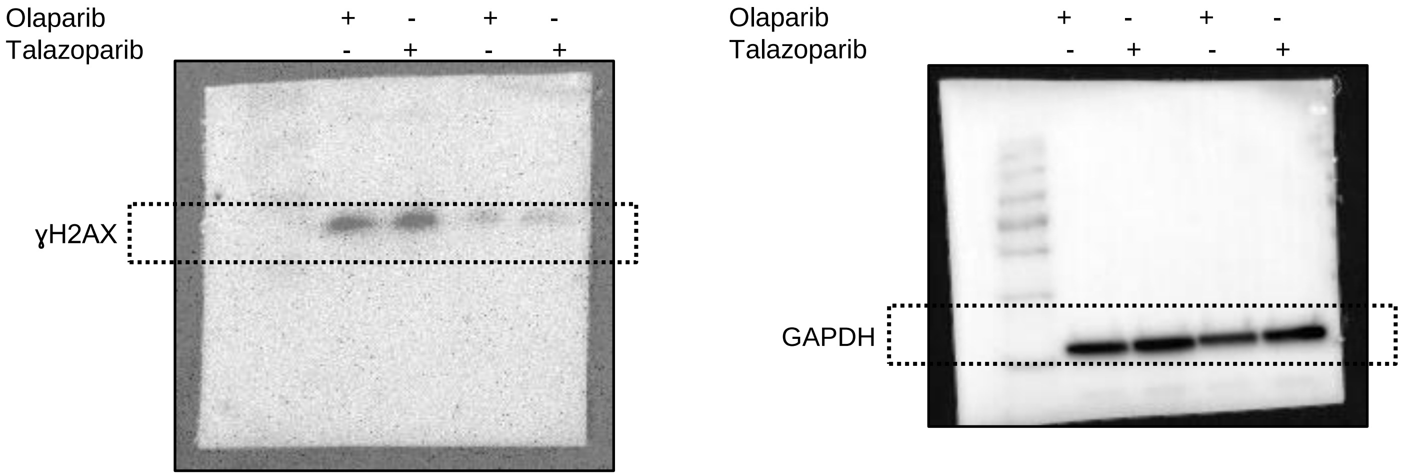

BT-474

I

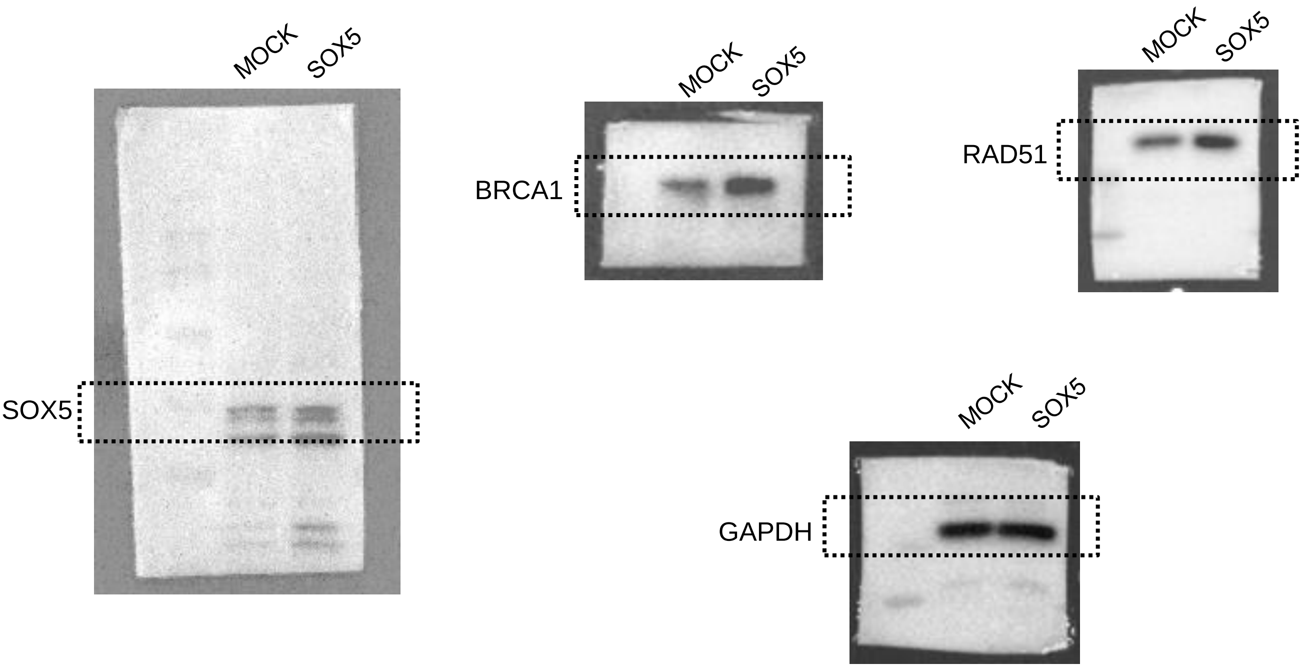

Fig 4

SNU251

J

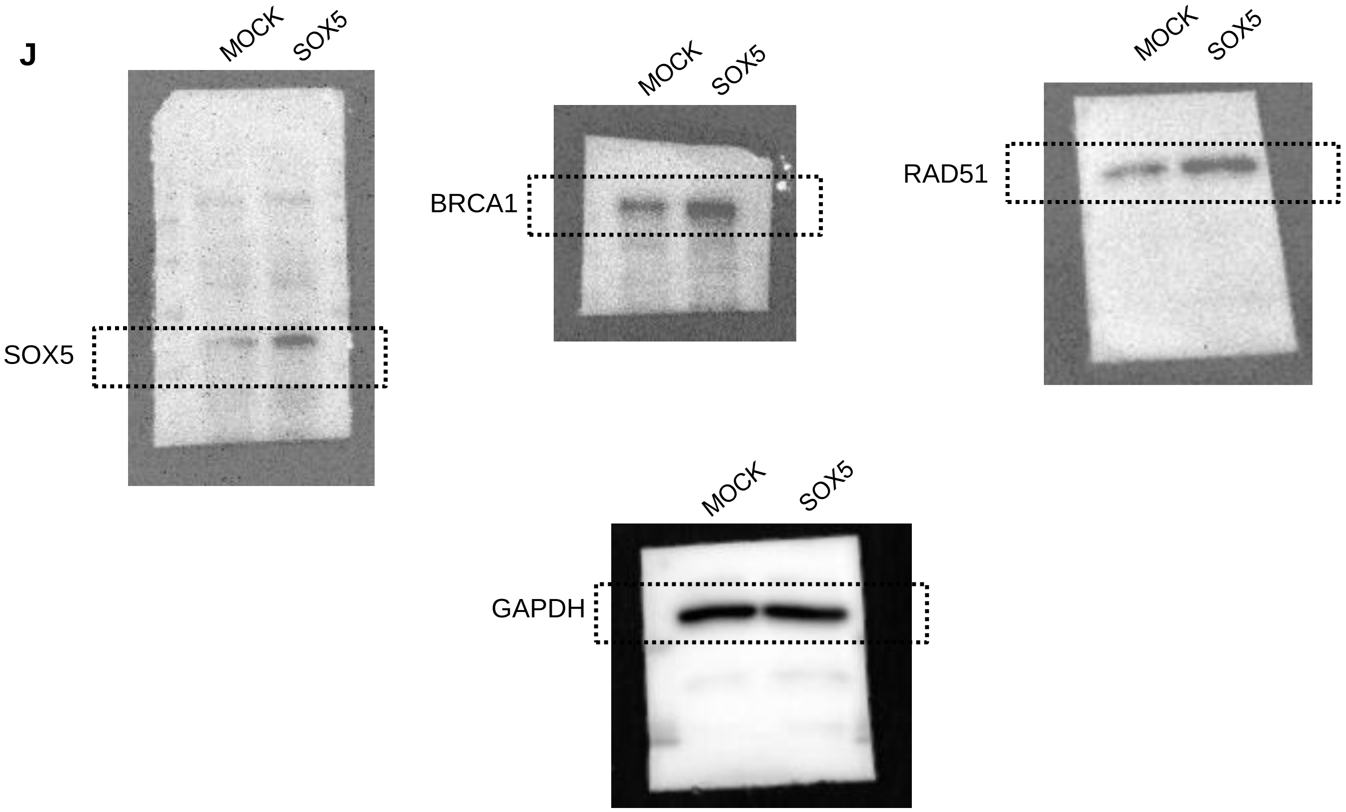

Fig 5

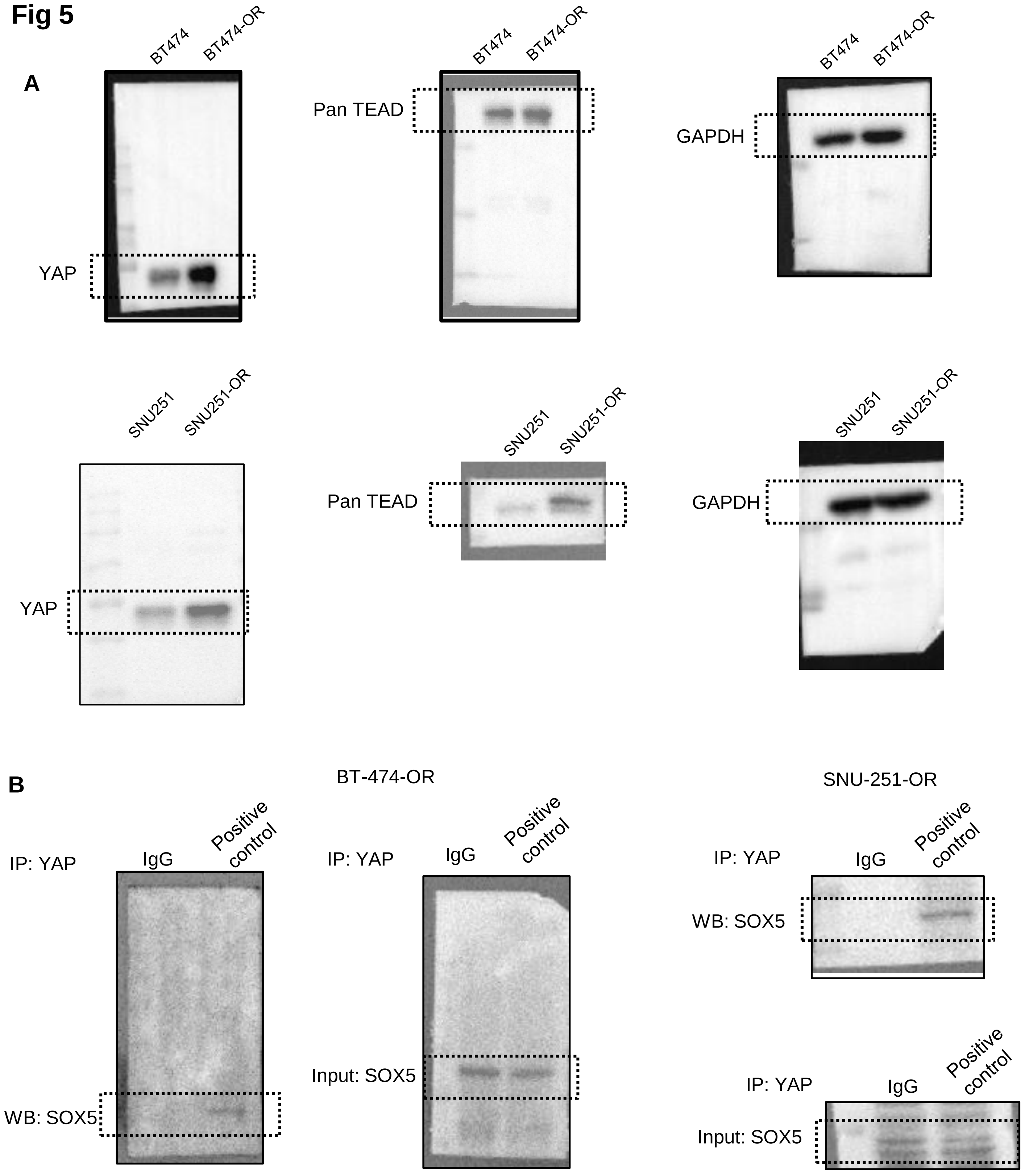
A
BT474
BT474-OR
YAP
Pan TEAD
GAPDH
SNU251
SNU251-OR
YAP
Pan TEAD
GAPDH
B
BT-474-OR
SNU-251-OR
IP: YAP
IgG
Positive control
WB: SOX5
Input: SOX5

Fig 5

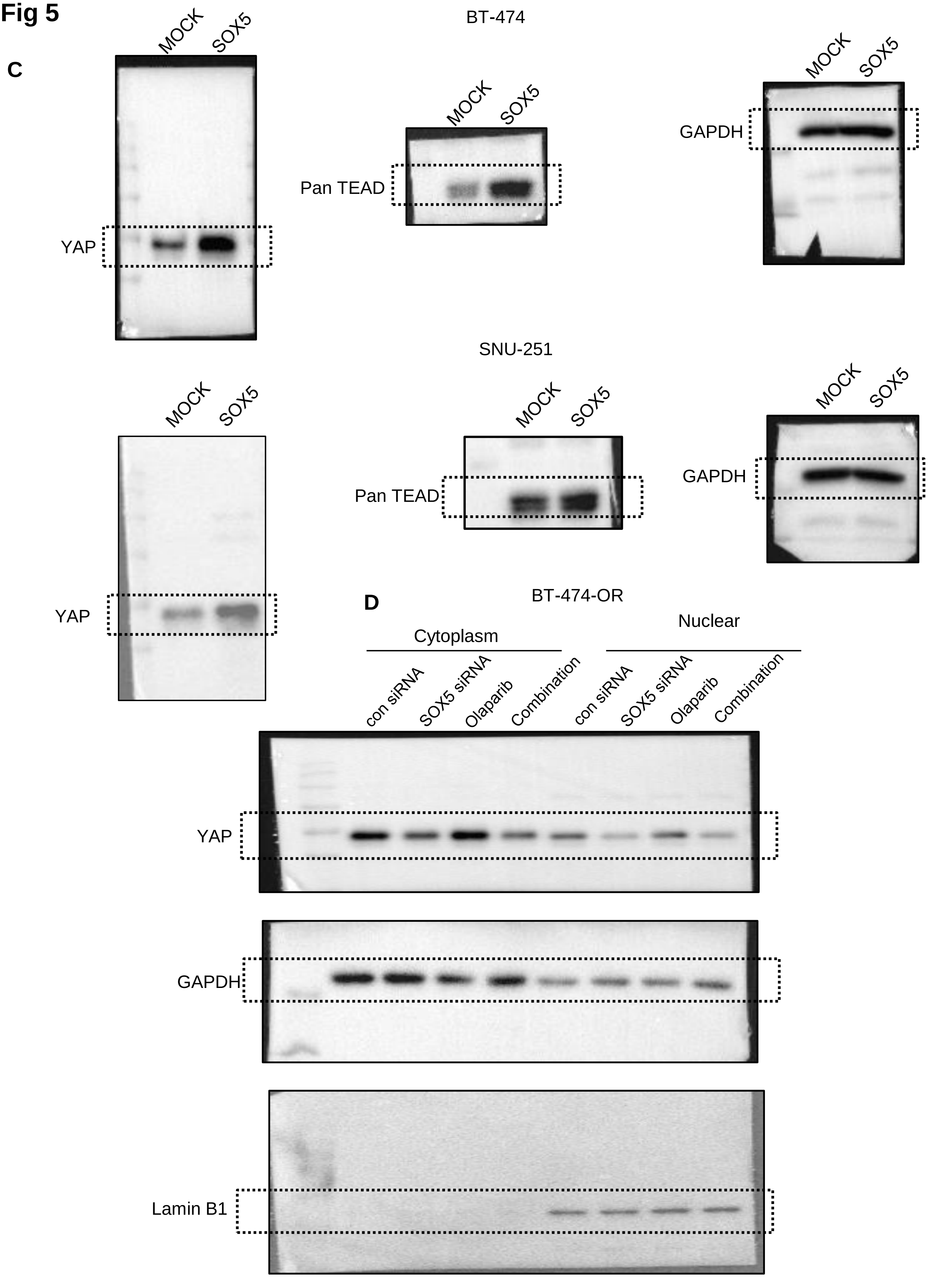
C
BT-474
MOCK
SOX5
YAP
Pan TEAD
GAPDH
SNU-251
MOCK
SOX5
YAP
Pan TEAD
GAPDH
D
BT-474-OR
Cytoplasm
Nuclear
con siRNA
SOX5 siRNA
Olaparib
Combination
con siRNA
SOX5 siRNA
Olaparib
Combination
YAP
GAPDH
Lamin B1

Fig 5

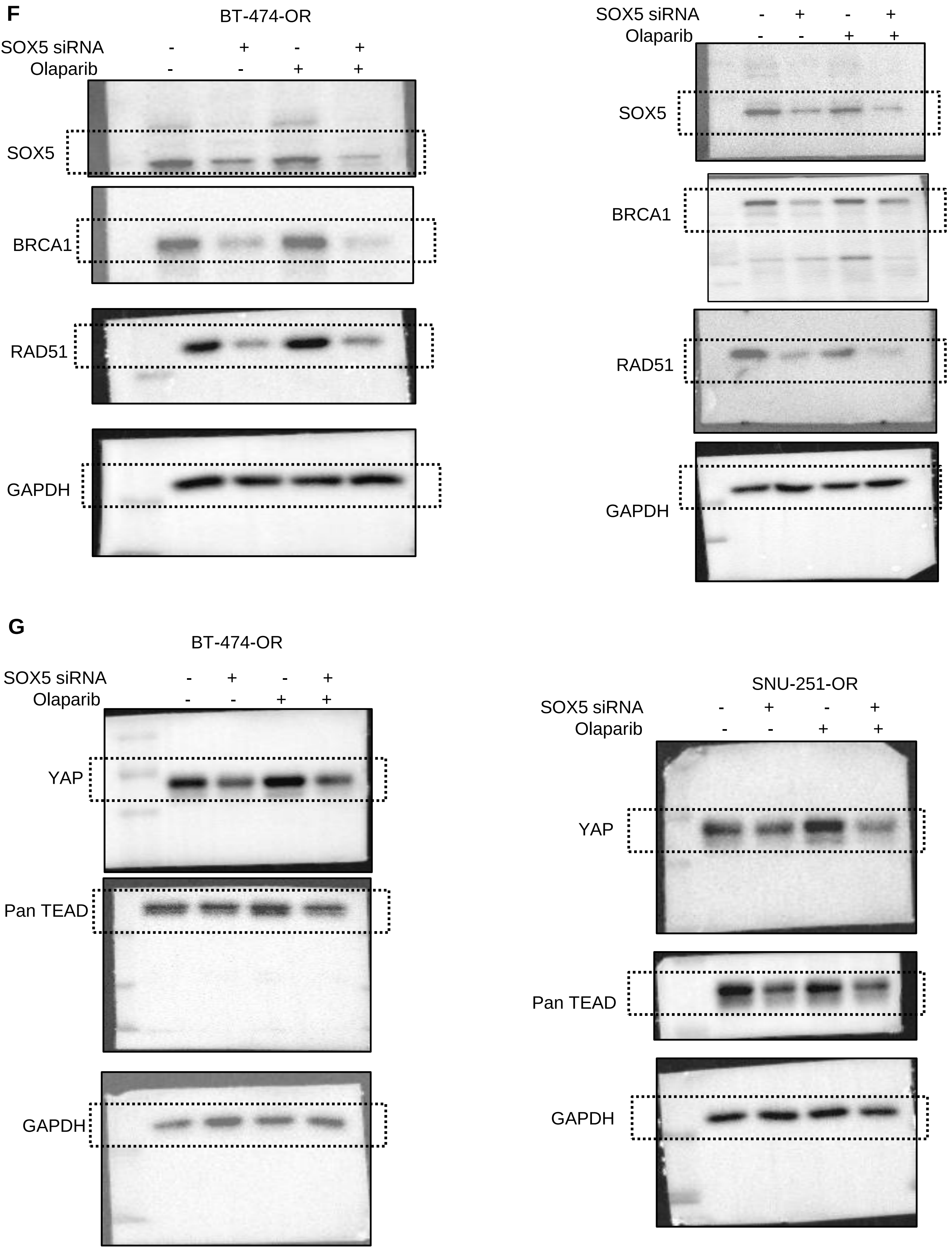
F
BT-474-OR
SOX5 siRNA - + - +
Olaparib - - + +
SOX5
BRCA1
RAD51
GAPDH
SNU-251-OR
SOX5 siRNA - + - +
Olaparib - - + +
SOX5
BRCA1
RAD51
GAPDH
G
BT-474-OR
SOX5 siRNA - + - +
Olaparib - - + +
YAP
Pan TEAD
GAPDH
SNU-251-OR
SOX5 siRNA - + - +
Olaparib - - + +
YAP
Pan TEAD
GAPDH

Fig 5

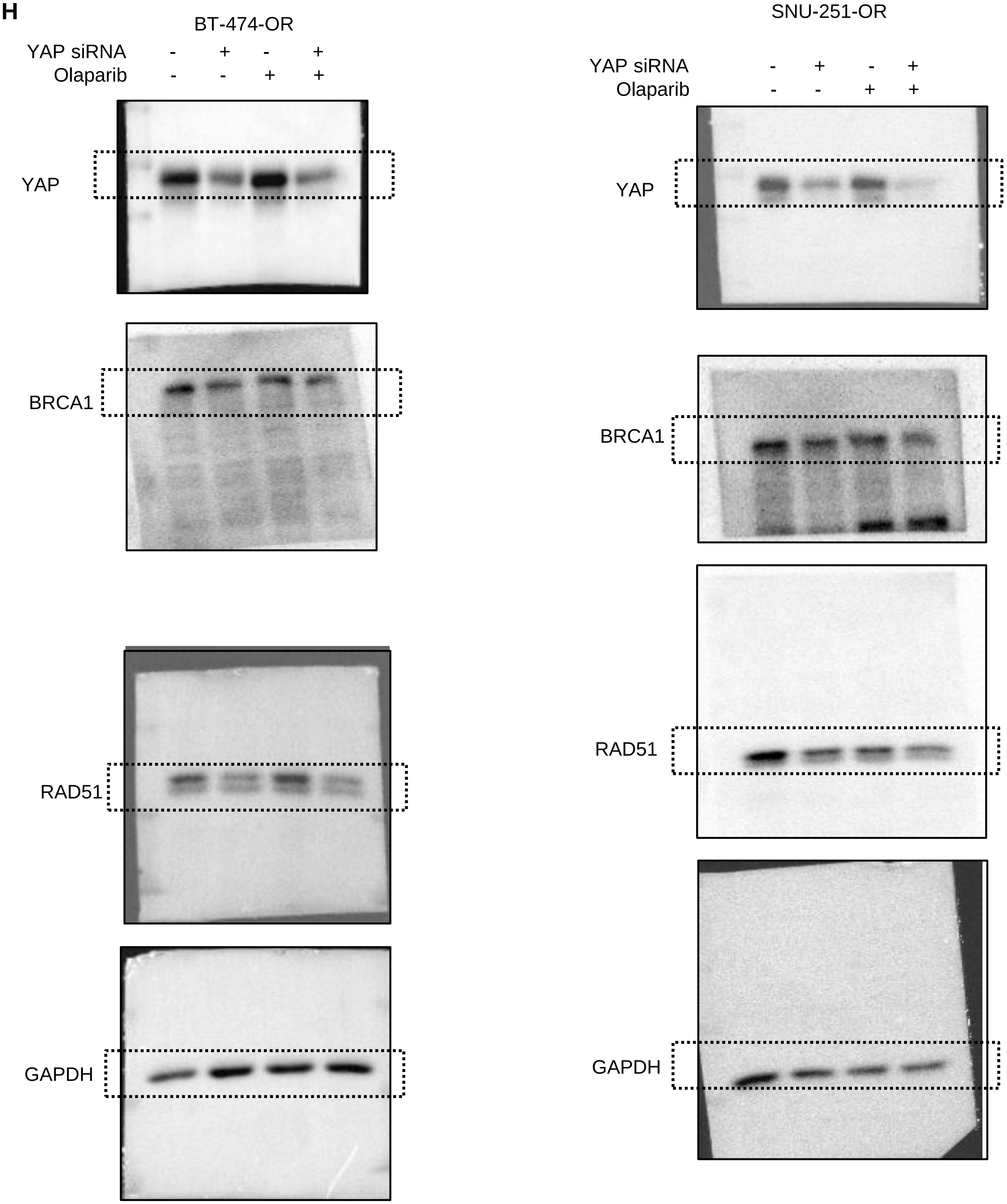
H
BT-474-OR
SNU-251-OR
YAP siRNA - + - +
Olaparib - - + +
YAP siRNA - + - +
Olaparib - - + +
YAP
BRCA1
RAD51
GAPDH
YAP
BRCA1
RAD51
GAPDH

Fig 6

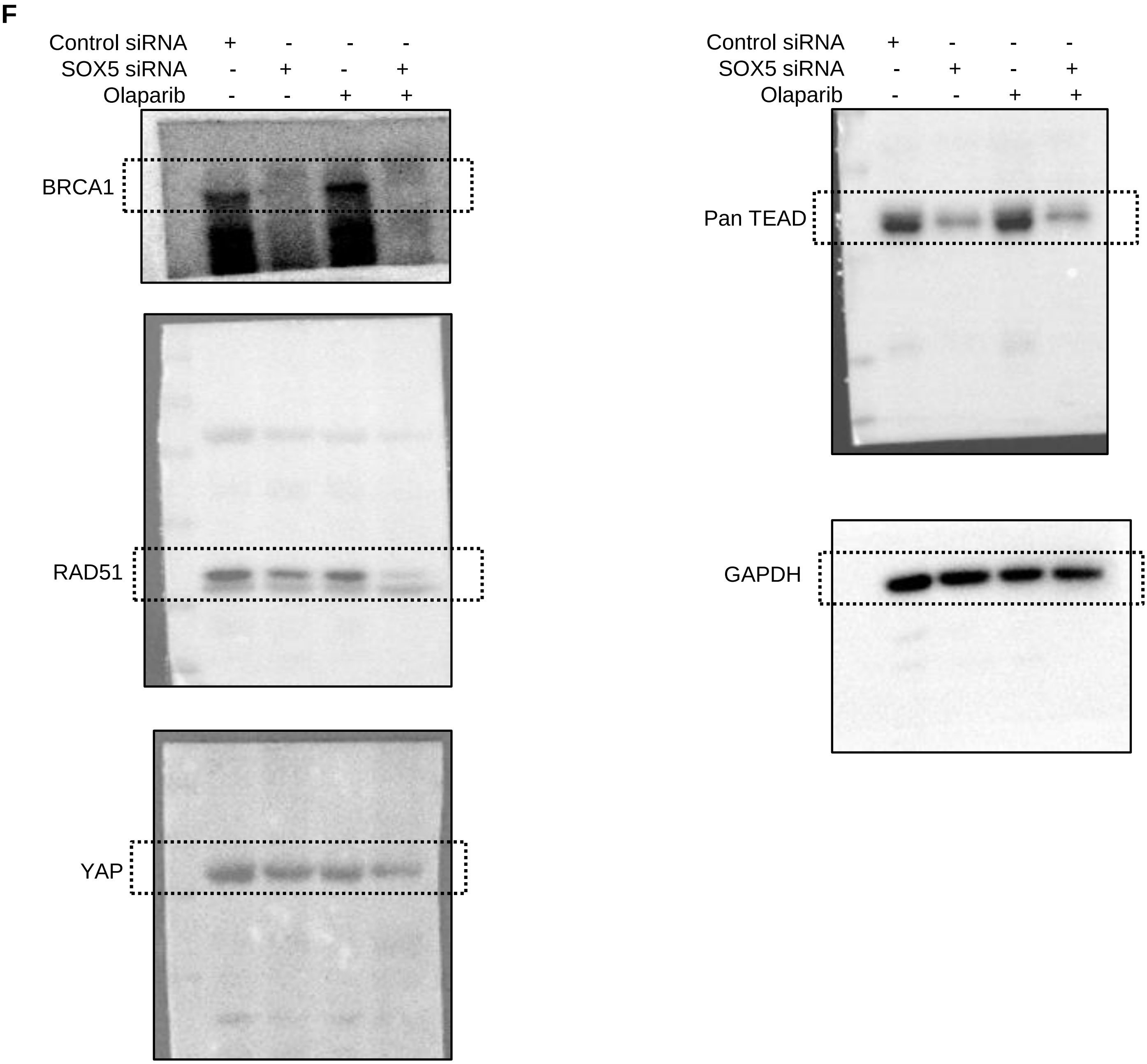
F
Control siRNA + - - -
SOX5 siRNA - + - +
Olaparib - - + +
BRCA1
RAD51
YAP
Control siRNA + - - -
SOX5 siRNA - + - +
Olaparib - - + +
Pan TEAD
GAPDH

Fig 6

G

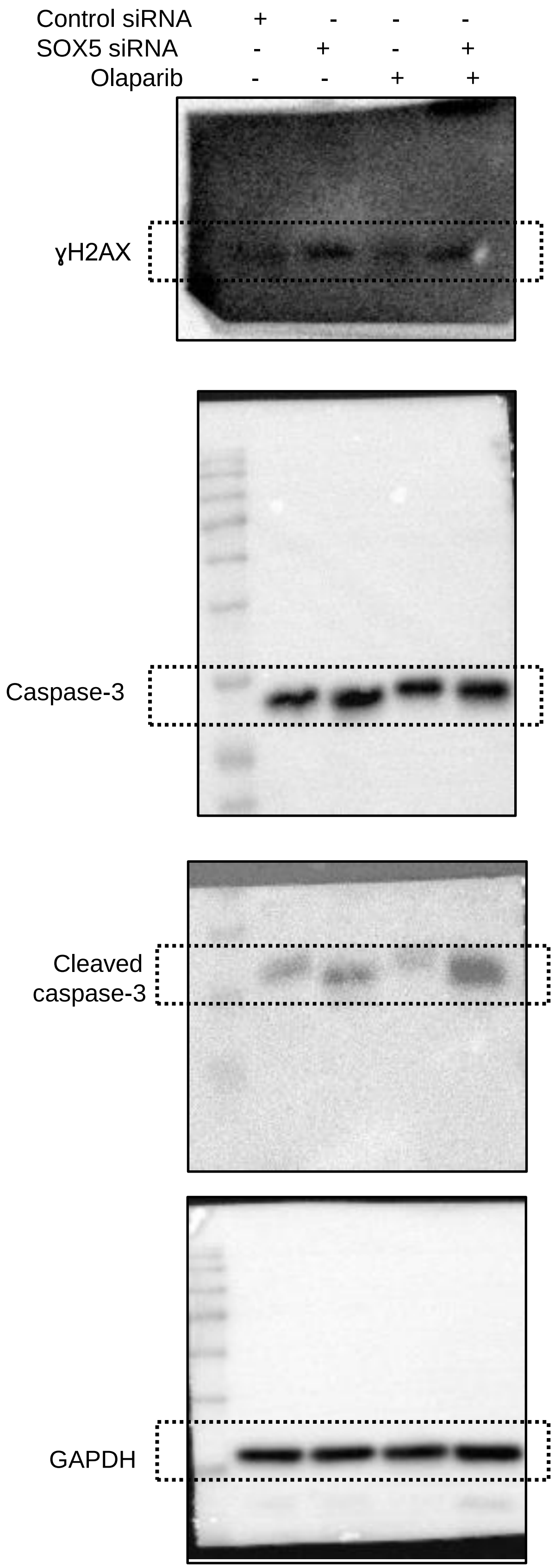

Control siRNA + - - -
SOX5 siRNA - + - +
Olaparib - - + +
ɣH2AX
Caspase-3
Cleaved caspase-3
GAPDH

Fig S2

G

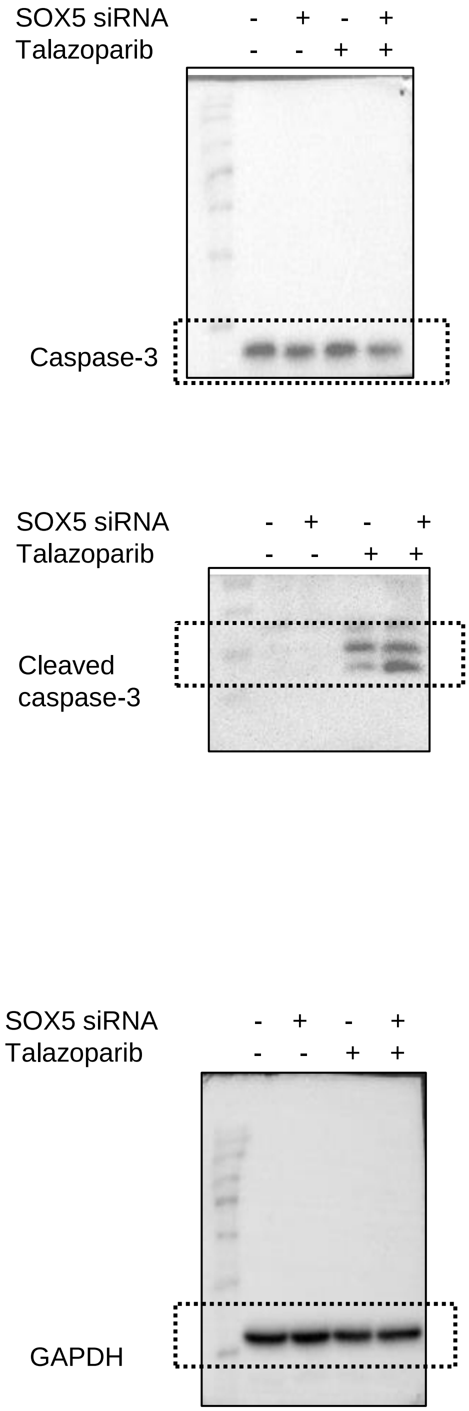
SOX5 siRNA - + - +
Talazoparib - - + +
Caspase-3
SOX5 siRNA - + - +
Talazoparib - - + +
Cleaved caspase-3
SOX5 siRNA - + - +
Talazoparib - - + +
GAPDH

H

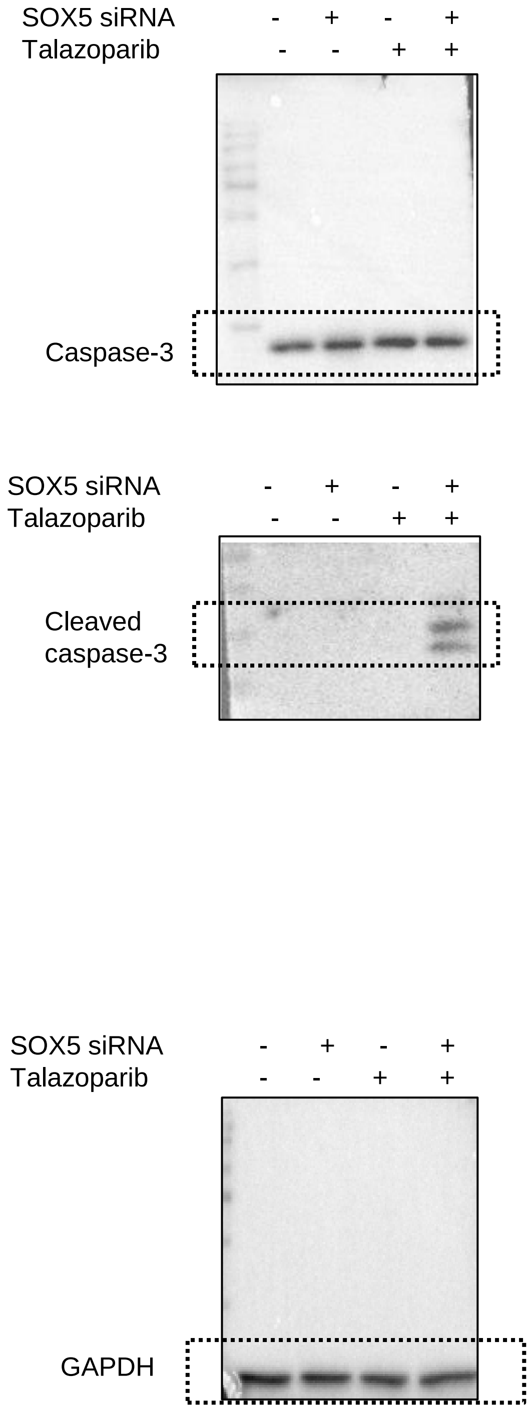
SOX5 siRNA - + - +
Talazoparib - - + +
Caspase-3
SOX5 siRNA - + - +
Talazoparib - - + +
Cleaved caspase-3
SOX5 siRNA - + - +
Talazoparib - - + +
GAPDH

Fig S4

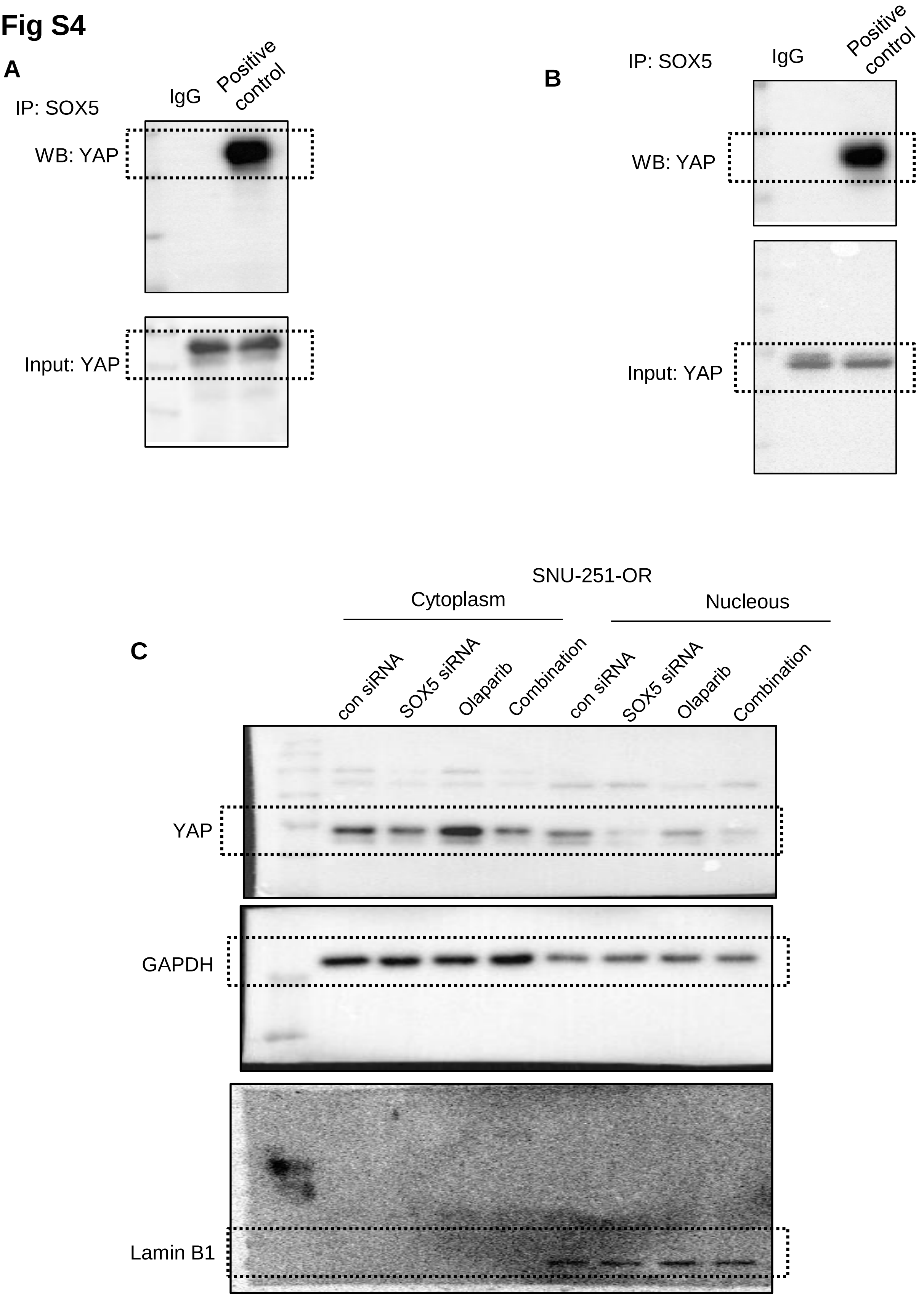
A
IP: SOX5
IgG
Positive control
WB: YAP
Input: YAP
B
IP: SOX5
IgG
Positive control
WB: YAP
Input: YAP
C
SNU-251-OR
Cytoplasm
Nucleous
con siRNA
SOX5 siRNA
Olaparib
Combination
con siRNA
SOX5 siRNA
Olaparib
Combination
YAP
GAPDH
Lamin B1

Fig S4

E

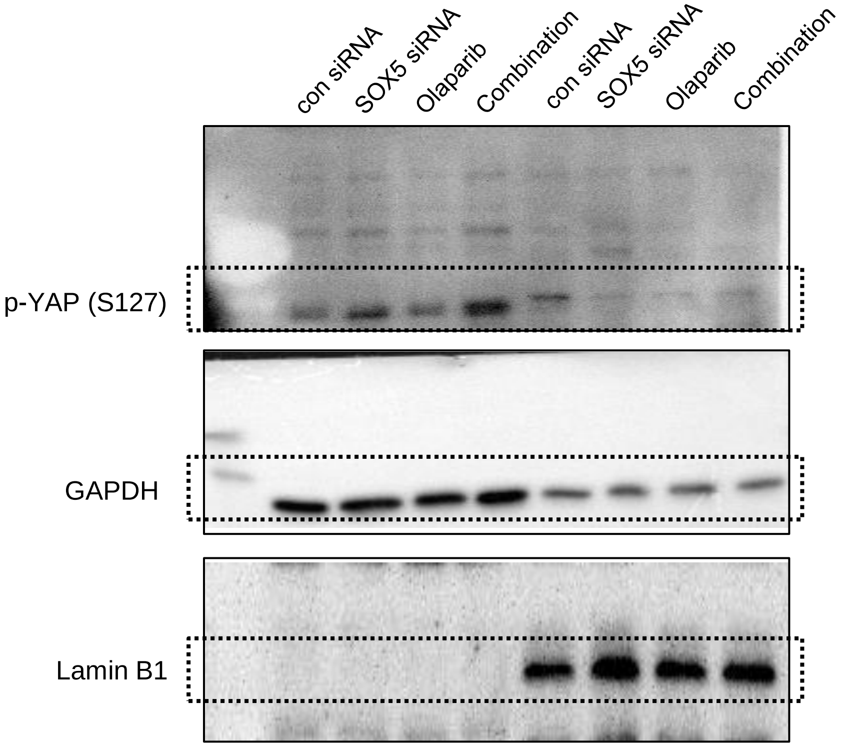
BT-474-OR
Cytoplasm
Nucleous
con siRNA
SOX5 siRNA
Olaparib
Combination
con siRNA
SOX5 siRNA
Olaparib
Combination
p-YAP (S127)
GAPDH
Lamin B1

F

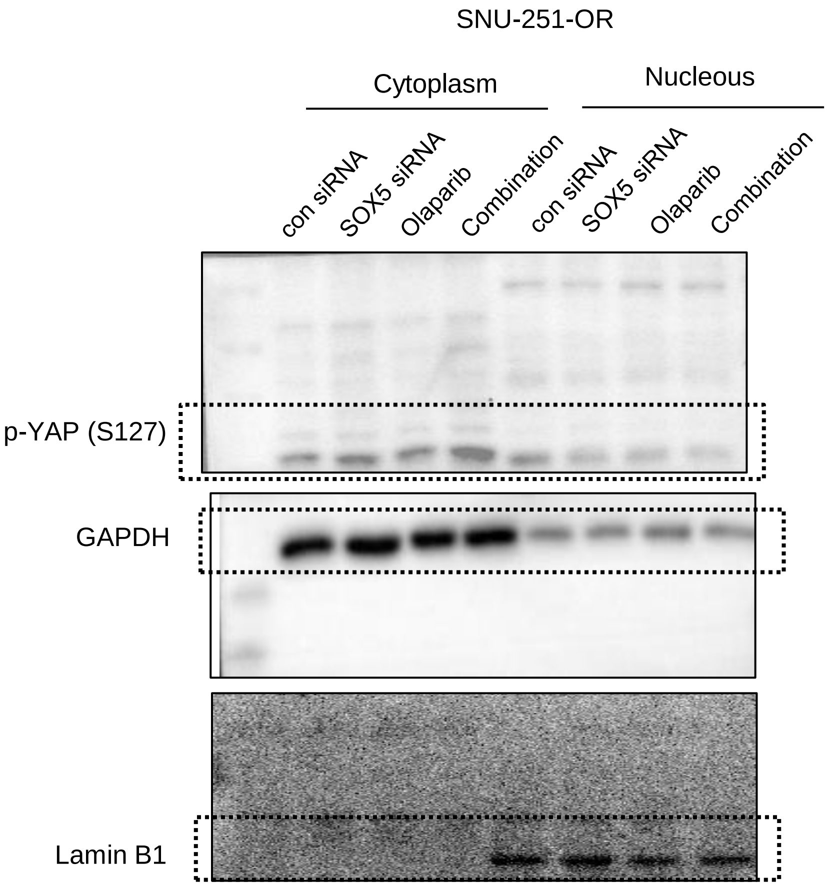
SNU-251-OR
Cytoplasm
Nucleous
con siRNA
SOX5 siRNA
Olaparib
Combination
con siRNA
SOX5 siRNA
Olaparib
Combination
p-YAP (S127)
GAPDH
Lamin B1

Fig S5

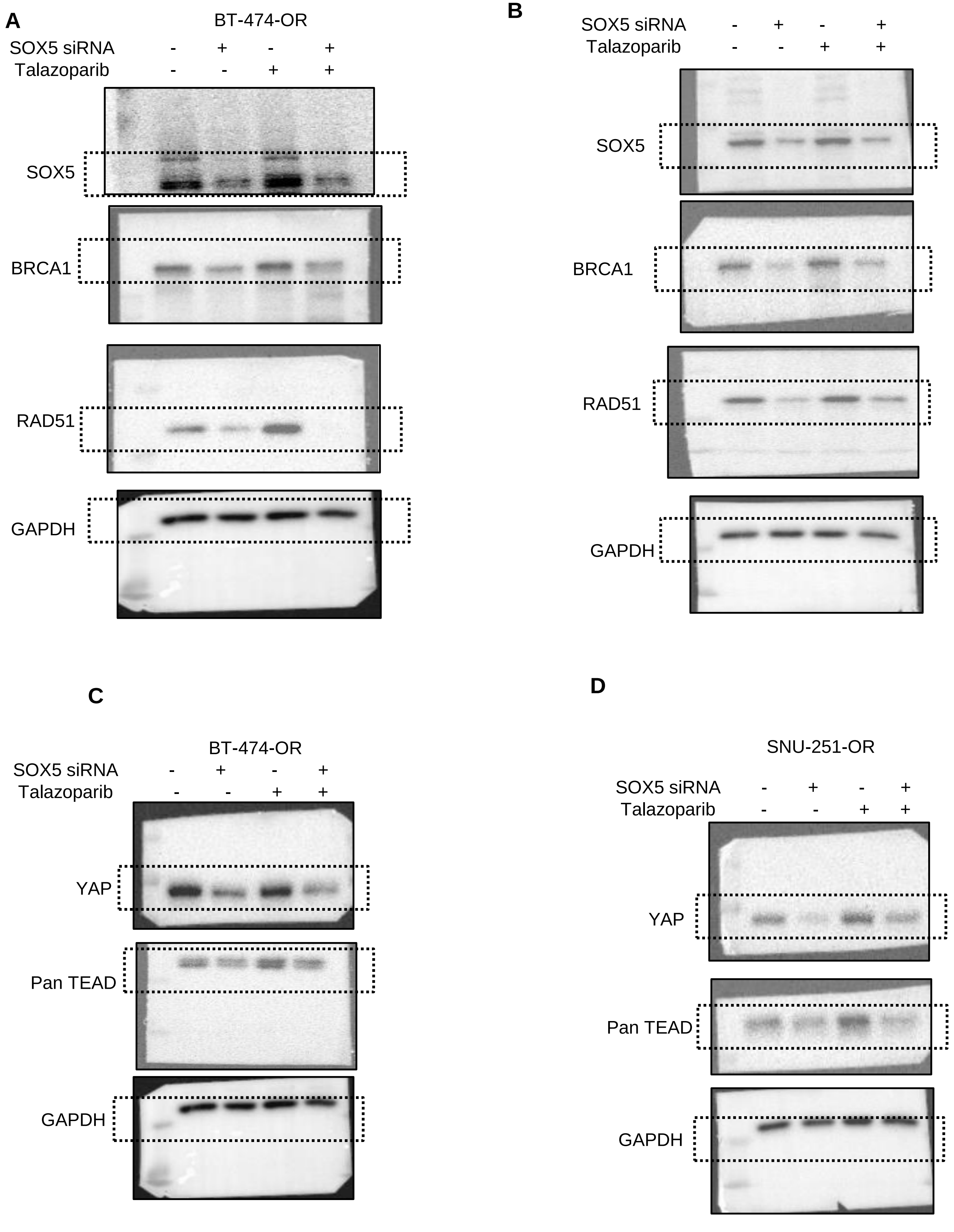
A
BT-474-OR
SOX5 siRNA - + - +
Talazoparib - - + +
SOX5
BRCA1
RAD51
GAPDH
B
SNU-251-OR
SOX5 siRNA - + - +
Talazoparib - - + +
SOX5
BRCA1
RAD51
GAPDH
C
BT-474-OR
SOX5 siRNA - + - +
Talazoparib - - + +
YAP
Pan TEAD
GAPDH
D
SNU-251-OR
SOX5 siRNA - + - +
Talazoparib - - + +
YAP
Pan TEAD
GAPDH

Fig S5

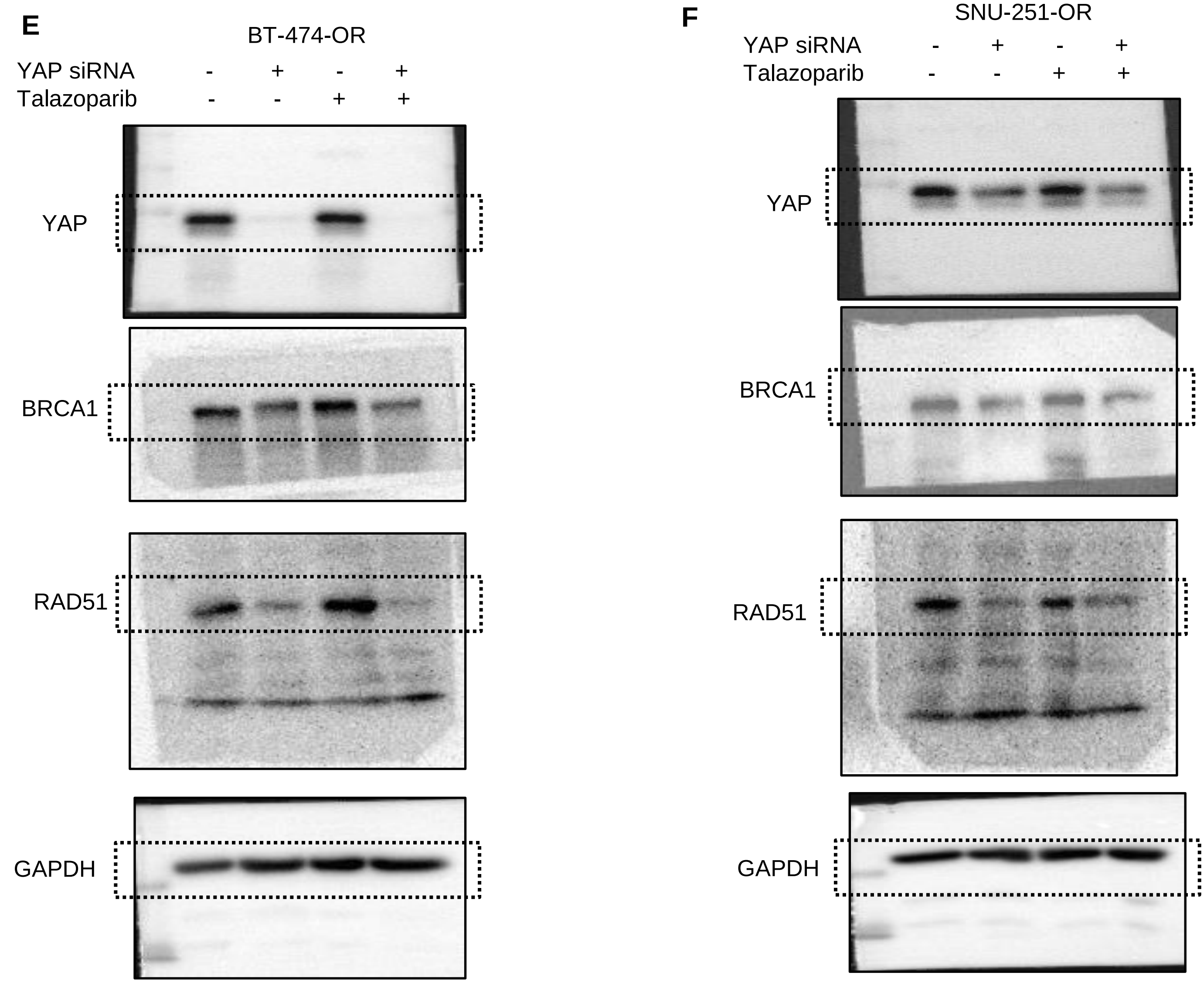
E
BT-474-OR
YAP siRNA - + - +
Talazoparib - - + +
YAP
BRCA1
RAD51
GAPDH
F
SNU-251-OR
YAP siRNA - + - +
Talazoparib - - + +
YAP
BRCA1
RAD51
GAPDH
